# Marjoram Extract Modulates Ion Currents, Intracellular Calcium Signaling, and MTT-Reducing Activity in Bovine Chromaffin Cells and Rat Cortical Neurons

**DOI:** 10.3390/ph19091493

**Published:** 2026-09-19

**Authors:** Ricardo de Pascual, María Arribas Tejedor, Sara Amor, Laura Jaime, Marisol Villalva, Susana Santoyo, Victoria Jimenez Carretero, Minerva Reyes Almodovar, Javier Hernández Campano, Miriam Granado, Jesús M. Hernández-Guijo

**Affiliations:** 1 Department of Pharmacology and Therapeutics, Facultad de Medicina, Universidad Autónoma de Madrid, Av. Arzobispo Morcillo 4, 28029 Madrid, Spain; ricardo.pascual@uam.es (R.d.P.); mariaarribas2000@gmail.com (M.A.T.); victoria.jimenez@uam.es (V.J.C.); minerva.reyes@uam.es (M.R.A.); esculebre@gmail.com (J.H.C.); 2 Department of Physiology, Facultad de Medicina, Universidad Autónoma de Madrid, C/Arzobispo Morcillo 4, 28029 Madrid, Spain; sara.amor@uam.es (S.A.); miriam.granado@uam.es (M.G.); 3 Department of Production and Characterization of Novel Foods, Food Science Research Institute (CIAL) (UAM + CSIC), Universidad Autónoma de Madrid, C/Nicolás Cabrera 9, 28049 Madrid, Spain; laura.jaime@uam.es (L.J.); marisol.villalva@ucm.es (M.V.); susana.santoyo@uam.es (S.S.); 4 Ramón y Cajal Institute for Health Research, IRYCIS, Hospital Ramón y Cajal, Ctra. de Colmenar Viejo, Km. 9100, 28029 Madrid, Spain

**Keywords:** *Origanum majorana*, bovine chromaffin cells, L-type calcium channels, ryanodine-sensitive stores, intracellular calcium, MTT-reducing activity, cortical neurons

## Abstract

**Background/Objectives: ***Origanum majorana* L. has reported antioxidant and other bioactive properties, but its effects on excitability and intracellular Ca^2+^ handling in excitable cells remain poorly defined. We investigated how a hydroalcoholic marjoram extract modulates membrane ion currents, cytosolic Ca^2+^ signaling, and MTT-reducing activity in bovine chromaffin cells and primary rat cortical neurons. **Methods:** A pressurized liquid *O. majorana* extract (ethanol:water, 70:30) was chemically characterized by HPLC-PAD. Whole-cell patch-clamp and current-clamp recordings were used to assess voltage-dependent Ca^2+^, Na^+^, and K^+^ currents and membrane excitability in bovine chromaffin cells. Fluo-4-AM measurements were used to evaluate Ca^2+^ responses to K^+^, caffeine, and histamine. MTT assays assessed changes in MTT-reducing activity under veratridine-induced Ca^2+^ overload and oligomycin/rotenone-induced mitochondrial stress in bovine chromaffin cells and cortical neurons prepared from embryonic day 18 Sprague-Dawley rats. For inferential statistics, replicate cells or wells from the same primary culture were averaged and the independent culture was used as the biological unit. **Results:** Culture-level analysis confirmed significant reductions in voltage-dependent Ca^2+^ and Na^+^ currents at 1.5 µg/mL (1 µL/mL) marjoram extract, together with significant overall effects on the putative Ca^2+^-dependent and voltage-dependent K^+^ current components. The extract depolarized the resting membrane potential and markedly suppressed repetitive action-potential firing. Mean K^+^-evoked Fluo-4 responses were reduced by approximately 20%, although this effect did not reach statistical significance at the culture level. Caffeine-evoked Ca^2+^ responses were higher in the presence of 0.015–1.5 µg/mL (0.01–1 µL/mL) marjoram extract, but these differences did not remain significant after correction for multiple comparisons. Histamine-evoked responses were unchanged. In MTT assays, significant preservation of MTT-reducing activity was limited to selected conditions, mainly at lower extract concentrations in bovine chromaffin cells, whereas the neuronal comparisons did not remain significant after multiplicity correction. Because extract-only, concentration-matched vehicle, cell-free extract/MTT interference controls, and an independent orthogonal assay of cell number or membrane integrity were not included, the MTT findings are reported strictly as changes in MTT-reducing activity and not as direct evidence of cytoprotection or neuroprotection. **Conclusions:** *O. majorana* extract modulates membrane excitability through effects on multiple plasma-membrane ion currents. The higher mean caffeine-evoked Ca^2+^ responses are compatible with altered Ca^2+^ mobilization from ryanodine-sensitive intracellular stores, but they do not demonstrate direct activation or sensitization of RyR receptors. The MTT findings do not establish cytoprotection or neuroprotection. These findings are correlative, and a causal contribution of ryanodine-sensitive intracellular stores to the preservation of MTT-reducing cellular metabolic activity remains to be established.

## 1. Introduction

Natural products obtained from medicinal and aromatic plants have long been considered a valuable source of bioactive molecules with pharmacological potential. Among them, essential oils and plant extracts from species of the Lamiaceae family have attracted particular interest because of their antioxidant, anti-inflammatory, antimicrobial, antispasmodic and cytoprotective activities. These biological properties are generally attributed to complex mixtures of secondary metabolites, including monoterpenes, sesquiterpenes, phenolic acids and flavonoids, which may act on multiple cellular targets either individually or synergistically [1,2].

*Origanum majorana* L., commonly known as sweet marjoram, is an aromatic and medicinal plant widely distributed in Mediterranean regions and traditionally used for the treatment of several disorders, including respiratory infections, gastrointestinal discomfort, intestinal spasms, hypertension, diabetes, insomnia and inflammatory conditions [1]. Previous pharmacological studies have supported many of these traditional uses, reporting antioxidant, antimicrobial, anti-inflammatory, antidiabetic, hepatoprotective, nephroprotective, antimutagenic, anticancer and gastrointestinal effects for different preparations of *O. majorana* [1,3,4]. Toxicological evaluations have also suggested a favorable safety profile, supporting further investigation of its pharmacodynamic mechanisms [1].

The chemical composition of *O. majorana* is variable and depends on geographical origin, cultivation conditions, extraction procedure and plant chemotype. Nevertheless, several studies consistently identify oxygenated monoterpenes as major constituents, particularly terpinen-4-ol, sabinene hydrate, linalool, linalyl acetate and α-terpineol [2,5,6]. These compounds, together with phenolic constituents such as rosmarinic acid, luteolin and apigenin derivatives, arbutin and hesperetin, are thought to contribute to the biological activity of marjoram preparations [7,8,9]. For instance, extracts of *Origanum majorana* from different geographical origins have shown relevant antioxidant and antimicrobial activities, while phytochemical studies have isolated phenolic compounds with antioxidant, antiproliferative and antibiofilm effects [5,7,8].

In recent years, increasing attention has been paid to the possible neuroprotective potential of *O. majorana*. Experimental studies have shown that marjoram extracts may protect against oxidative stress, neuroinflammation and cognitive impairment. In a murine model of lipopolysaccharide-induced neuroinflammation, *O. majorana* hydroalcoholic extract improved recognition and spatial memory, reduced histological signs of neurodegeneration, and attenuated astrocyte activation and COX-2 expression [9]. Similarly, *O. majorana* essential oil improved memory performance, inhibited acetylcholinesterase activity and reduced oxidative stress markers in a zebrafish model of scopolamine-induced cognitive impairment [6].

Clinical pilot studies in patients with idiopathic Parkinson’s disease have also suggested that *O. majorana* tea consumption may improve non-motor symptoms and depression scores, although motor symptoms were not significantly modified [10]. A related randomized placebo-controlled study further reported that marjoram tea enhanced antioxidant status, increasing superoxide dismutase and catalase activities while reducing carbonylated protein levels [11]. These observations are consistent with the idea that marjoram may exert beneficial effects in neurodegenerative conditions possibly through antioxidant and anti-inflammatory mechanisms [6,9,10,11].

Calcium homeostasis is a central determinant of neuronal and neuroendocrine cell function. Cytosolic calcium signals regulate excitability, secretion, mitochondrial activity, gene expression and cell survival. Alterations in calcium entry through voltage-dependent calcium channels or calcium release from intracellular stores may contribute to excitotoxicity, oxidative stress and cell death. Therefore, compounds able to modulate calcium handling may have important functional consequences in excitable cells. In this context, previous studies have proposed that *O. majorana* extracts can behave as calcium-channel inhibitory agents in gastrointestinal smooth muscle models. Aqueous or methanolic extracts of *O. majorana* relaxed intestinal preparations, shifted calcium concentration–response curves, and showed verapamil-like effects, suggesting inhibition of voltage-dependent calcium entry as one mechanism underlying its antispasmodic and antidiarrheal activity [3,12].

Despite this evidence, the direct effects of *O. majorana* on the electrical excitability of neurosecretory cells and on the mechanisms controlling intracellular calcium mobilization remain poorly understood. Bovine adrenal chromaffin cells represent a well-established model for studying stimulus–secretion coupling, voltage-dependent ion channels, cytosolic calcium dynamics and catecholamine release. These cells express voltage-dependent calcium, sodium and potassium channels and share several functional properties with sympathetic neurons, making them particularly useful to investigate how natural compounds modulate excitability and calcium-dependent cellular responses [13,14,15,16].

Based on this background, the present study was designed to characterize the effects of *O. majorana* extract on bovine chromaffin cells and primary rat cortical neurons. We analyzed its actions on voltage-dependent calcium, sodium and potassium currents, membrane excitability, cytosolic calcium elevations induced by different stimuli, and MTT-reducing activity under toxic conditions. Special attention was paid to distinguishing the effects of marjoram on calcium entry through plasma membrane channels from its possible influence on intracellular calcium mobilization through ryanodine-sensitive or IP_3_-sensitive stores. This approach allows us to examine whether these cellular effects are associated with changes in MTT-reducing activity under veratridine- or oligomycin/rotenone-induced stress without treating the MTT endpoint as a direct measure of cytoprotection or neuroprotection.

## 2. Results

### 2.1. Composition of Phenolic Compounds in the Marjoram Extract

In this study, a pressurized liquid extract from *O. majorana* was obtained using ethanol:water (70:30), as previously described [17]. The phenolic profile comprised 37 quantified compounds, 18 of which were tentatively identified on the basis of their UV–Vis spectra and MS/MS fragmentation patterns, according to previously established criteria [17,18]. The extract was particularly rich in flavones and phenolic acid derivatives. The most abundant compounds were 6-hydroxyluteolin-7-*O*-glucoside (40.34 ± 3.07 mg/g extract), rosmarinic acid (36.17 ± 1.40 mg/g extract), lithospermic acid isomer I (22.14 ± 0.71 mg/g extract), luteolin-*O*-glucoside (21.97 ± 1.55 mg/g extract), and luteolin-7-*O*-glucoside (16.78 ± 0.94 mg/g extract) (Table 1). This phenolic profile is consistent with previous studies reporting luteolin and apigenin derivatives, together with rosmarinic acid and related phenolic compounds, as characteristic constituents of marjoram extracts [18,19,20]. The technical specifications of the marjoram leaves used in this study, including their physicochemical, microbiological, nutritional, and allergen characteristics, are provided in Supplementary Material S1.

### 2.2. Marjoram Extract Partially Inhibits Voltage-Dependent Calcium Currents in Bovine Chromaffin Cells

**Figure 1:**
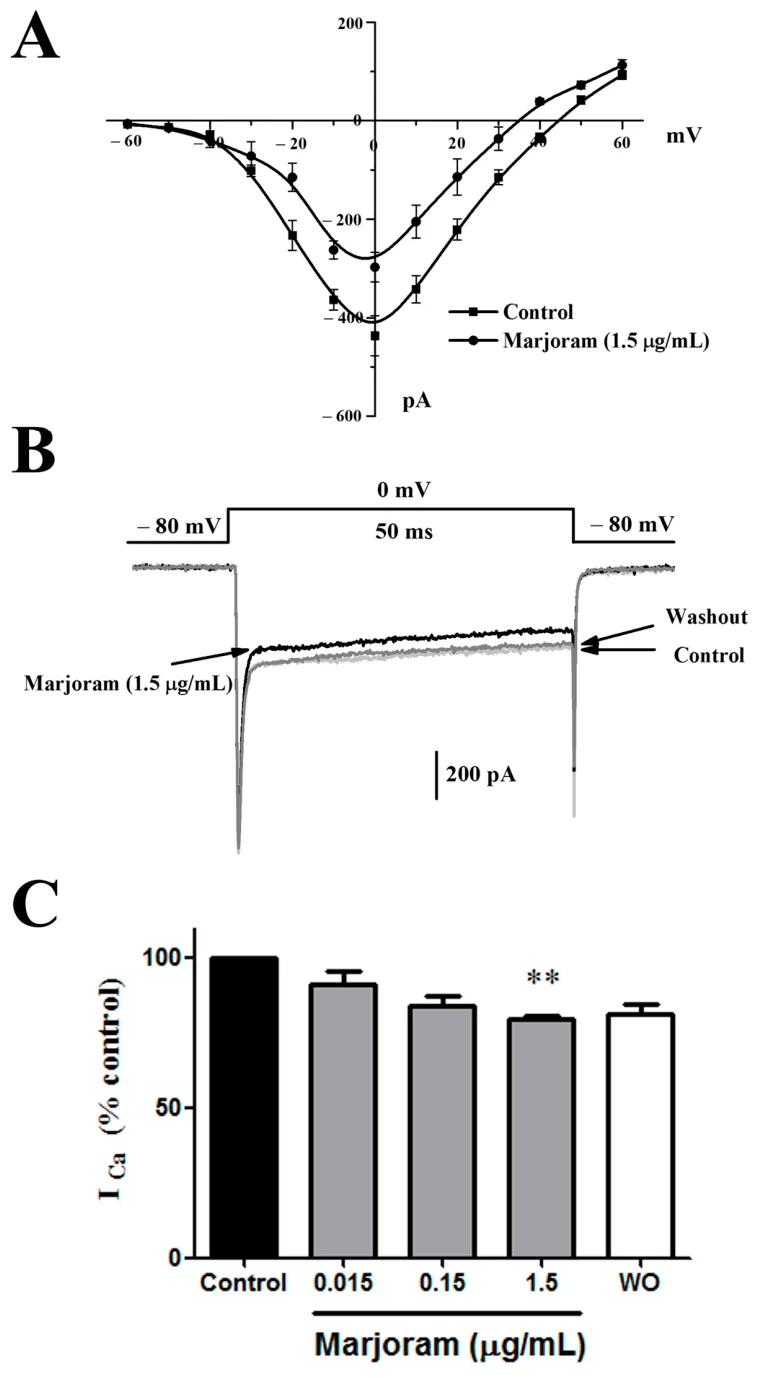
Marjoram extract partially inhibits voltage-dependent Ca^2+^ currents in bovine chromaffin cells. (**A**) Current–voltage relationship of whole-cell inward Ca^2+^ currents recorded under control conditions and in the presence of marjoram extract (1.5 µg/mL). Depolarizing test pulses were applied from a holding potential of −80 mV to the indicated test potentials. (**B**) Representative Ca^2+^ current traces obtained under control conditions, during application of marjoram extract (1.5 µg/mL), and after washout. Currents were evoked by 50 ms depolarizing pulses from −80 to 0 mV at 30 s intervals; the voltage protocol is shown above panel (**B**). (**C**) Concentration-dependent effect of marjoram extract on normalized peak I_Ca_, expressed as percentage of the initial control amplitude. A total of n = 9 cells from N = 4 independent cultures were analyzed. Recordings from cells belonging to the same culture were averaged before inferential analysis. Because the concentration series constituted a within-culture repeated-measures design, a one-way repeated-measures ANOVA was used, followed by Holm-adjusted prespecified comparisons with control: 0.015 µg/mL, *p* = 0.259; 0.15 µg/mL, *p* = 0.088; and 1.5 µg/mL, ** *p* = 0.0076. Data are presented as mean ± SEM of culture-level values; at 1.5 µg/mL, the mean paired difference was −20.7 percentage points (95% CI, −27.7 to −13.7; Cohen’s dz = 4.71). Volumetric stock equivalents are defined in Section 4.2. Here, n denotes individual cells and N denotes independent primary cultures.

We first investigated whether acute application of *Origanum majorana* extract modified voltage-dependent Ca^2+^ currents (*I*_Ca_) in bovine chromaffin cells. To obtain the current–voltage (I–V) relationship shown in Figure 1A, whole-cell inward Ca^2+^ currents were elicited by 50 ms depolarizing pulses from a holding potential of −80 mV to the indicated test potentials. Under control conditions, the I–V relationship displayed the characteristic profile of voltage-dependent Ca^2+^ currents, with maximal inward current around 0 mV (436.9 ± 40.9 pA). In the presence of marjoram extract 1.5 µg/mL (1 µL/mL), the mean current amplitude at 0 mV was lower (297.3 ± 70.3 pA), without an evident change in the overall shape of the I–V relationship.

For the time-course and concentration–response experiments, currents were evoked by 50 ms depolarizing pulses from −80 to 0 mV at 30 s intervals. Marjoram extract was applied after current stabilization. As shown by the representative traces in Figure 1B, marjoram extract at 1.5 µg/mL reduced peak *I_Ca_*, with partial recovery after washout. In Figure 1C, peak *I*_Ca_ was normalized in each cell to its initial control amplitude (100%). Marjoram extract reduced *I*_Ca_ to 90.7 ± 2.6%, 86.7 ± 3.1%, and 79.3 ± 3.5% of control at 0.015, 0.15, and 1.5 µg/mL (0.01, 0.1, and 1 µL/mL), respectively. Because all concentrations were evaluated within the same independent cultures, the concentration series was treated as a repeated-measures design. After averaging recordings within each of the N = 4 cultures, a one-way repeated-measures ANOVA was followed by prespecified comparisons of each concentration with control, with Holm correction for multiplicity. The effects at 0.015 and 0.15 µg/mL were not significant after correction (adjusted *p* = 0.259 and *p* = 0.088, respectively), whereas the reduction at 1.5 µg/mL remained significant (adjusted *p* = 0.0076; mean paired difference, −20.7 percentage points; 95% CI, −27.7 to −13.7; Cohen’s dz = 4.71).

Taken together, these results indicate that marjoram extract partially inhibits voltage-dependent Ca^2+^ currents in bovine chromaffin cells, with the culture-level analysis supporting a significant effect at 1.5 µg/mL and a maximal mean inhibition of approximately 20%.

### 2.3. Marjoram Extract Preferentially Affects the L-Type Component of Voltage-Dependent Calcium Currents

**Figure 2:**
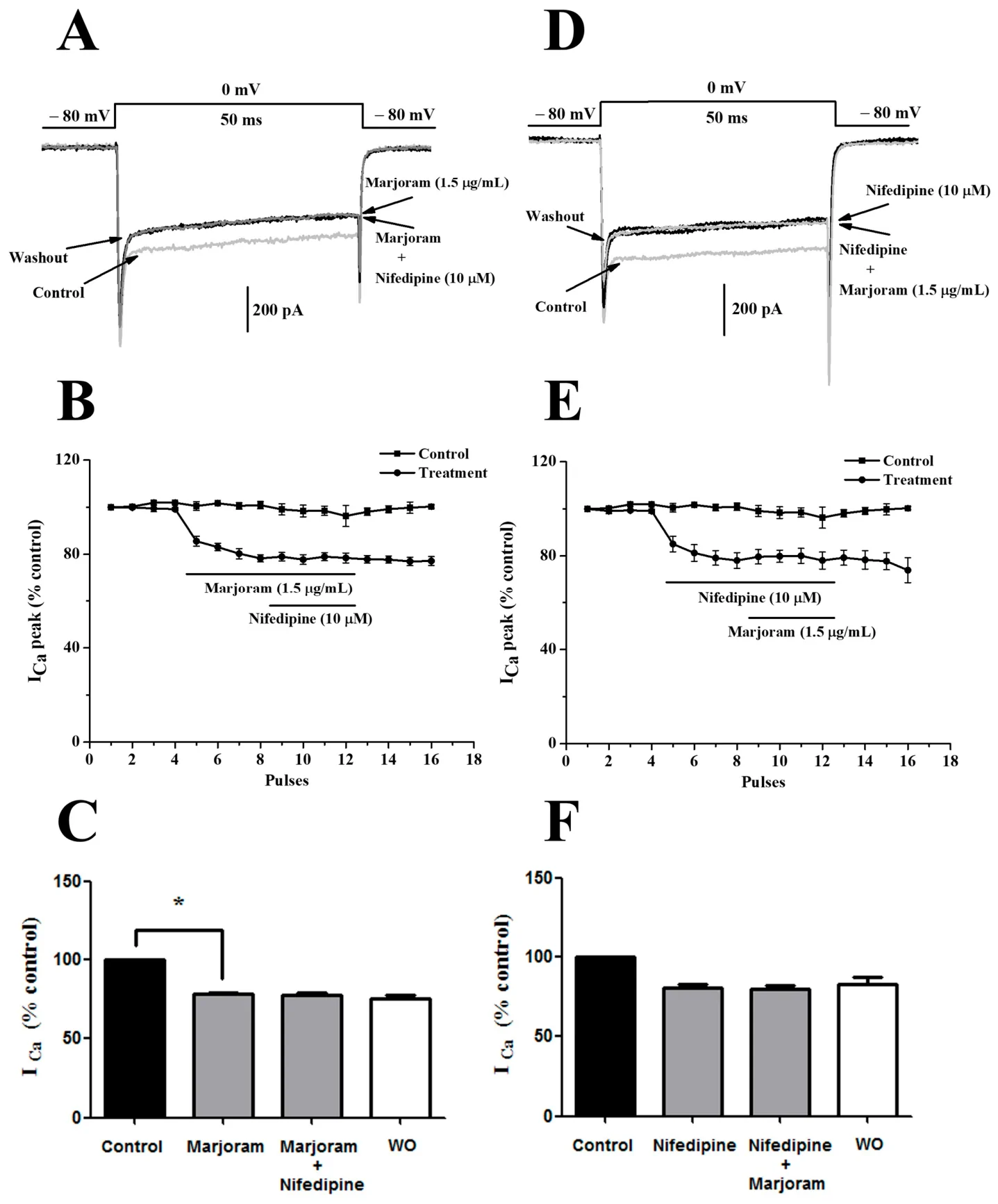
Marjoram extract preferentially affects the L-type component of voltage-dependent Ca^2+^ currents in bovine chromaffin cells. (**A**) Representative current traces obtained under control conditions, during application of marjoram extract (1.5 µg/mL), during marjoram extract plus nifedipine (10 µM), and after washout. (**B**) Time course of normalized peak *I*_Ca_ during sequential application of marjoram extract followed by nifedipine. (**C**) Culture-level summary for the protocol shown in (**A**,**B**). (**D**) Representative traces obtained with the reverse order of application. (**E**) Corresponding time course. (**F**) Culture-level summary for the protocol shown in (**D**,**E**). A total of n = 7 cells from N = 3 independent cultures were analyzed, and multiple cellular recordings from the same culture were averaged before statistical testing. Each sequence was analyzed by one-way repeated-measures ANOVA, followed by two prespecified paired contrasts with Holm correction. Forward sequence: control vs. marjoram, * *p* = 0.0166; marjoram vs. marjoram + nifedipine, *p* = 0.81. Reverse sequence: control vs. nifedipine, *p* = 0.072; nifedipine vs. nifedipine + marjoram, *p* = 0.90. These analyses test the incremental effect of the second treatment directly and avoid multiple uncorrected *t*-tests. Here, n denotes individual cells and N denotes independent primary cultures.

To determine whether the partial inhibition of the total voltage-dependent Ca^2+^ current produced by *Origanum majorana* extract involved the L-type channel component, we performed sequential pharmacological experiments using nifedipine, a selective blocker of L-type voltage-dependent Ca^2+^ channels. In the first protocol (Figure 2A–C), marjoram extract (1.5 µg/mL) was applied first and reduced peak *I*_Ca_ to 78.3 ± 1.3% of control. Subsequent addition of nifedipine (10 µM) in the continued presence of marjoram produced little further change, with *I*_Ca_ remaining at 77.5 ± 1.4% of control. Representative current traces and the corresponding time course are shown in Figure 2A,B.

The reverse sequence produced a similar pattern. When nifedipine (10 µM) was applied first, peak *I*_Ca_ was reduced to 80.5 ± 2.5% of control; subsequent application of marjoram extract (1.5 µg/mL) in the continued presence of nifedipine yielded a value of 79.7 ± 2.9% of control (Figure 2D–F). Representative traces and the time course of this protocol are shown in Figure 2D and Figure 2E, respectively. Thus, irrespective of the order of application, the second treatment produced no appreciable additional inhibition beyond the approximately 20% reduction produced by the first treatment.

Culture-level analysis was performed using N = 3 independent cultures (n = 7 cells), consistent with the experimental numbers shown in Figure 2. Each application sequence was analyzed separately as a within-culture repeated-measures design using one-way repeated-measures ANOVA. To avoid redundant multiple testing, two prespecified paired contrasts were evaluated in each sequence and corrected by the Holm procedure: (i) control versus the first treatment and (ii) the first treatment versus the same treatment after addition of the second agent. In the forward sequence, marjoram significantly reduced *ICa* compared with control (Holm-adjusted *p* = 0.0166), whereas subsequent addition of nifedipine produced no significant incremental change (marjoram vs. marjoram + nifedipine, adjusted *p* = 0.81). In the reverse sequence, the reduction produced by nifedipine did not remain significant after multiplicity correction (adjusted *p* = 0.072), and addition of marjoram produced no significant incremental effect (nifedipine vs. nifedipine + marjoram, adjusted *p* = 0.90).

Taken together, the absence of a significant incremental effect when the second treatment was added is consistent with preferential involvement of the dihydropyridine-sensitive L-type component in the effect of marjoram extract. These data support an overlapping action on the L-type component but do not demonstrate selective blockade of L-type channels.

### 2.4. Marjoram Extract Partially Inhibits Voltage-Dependent Sodium Currents

**Figure 3:**
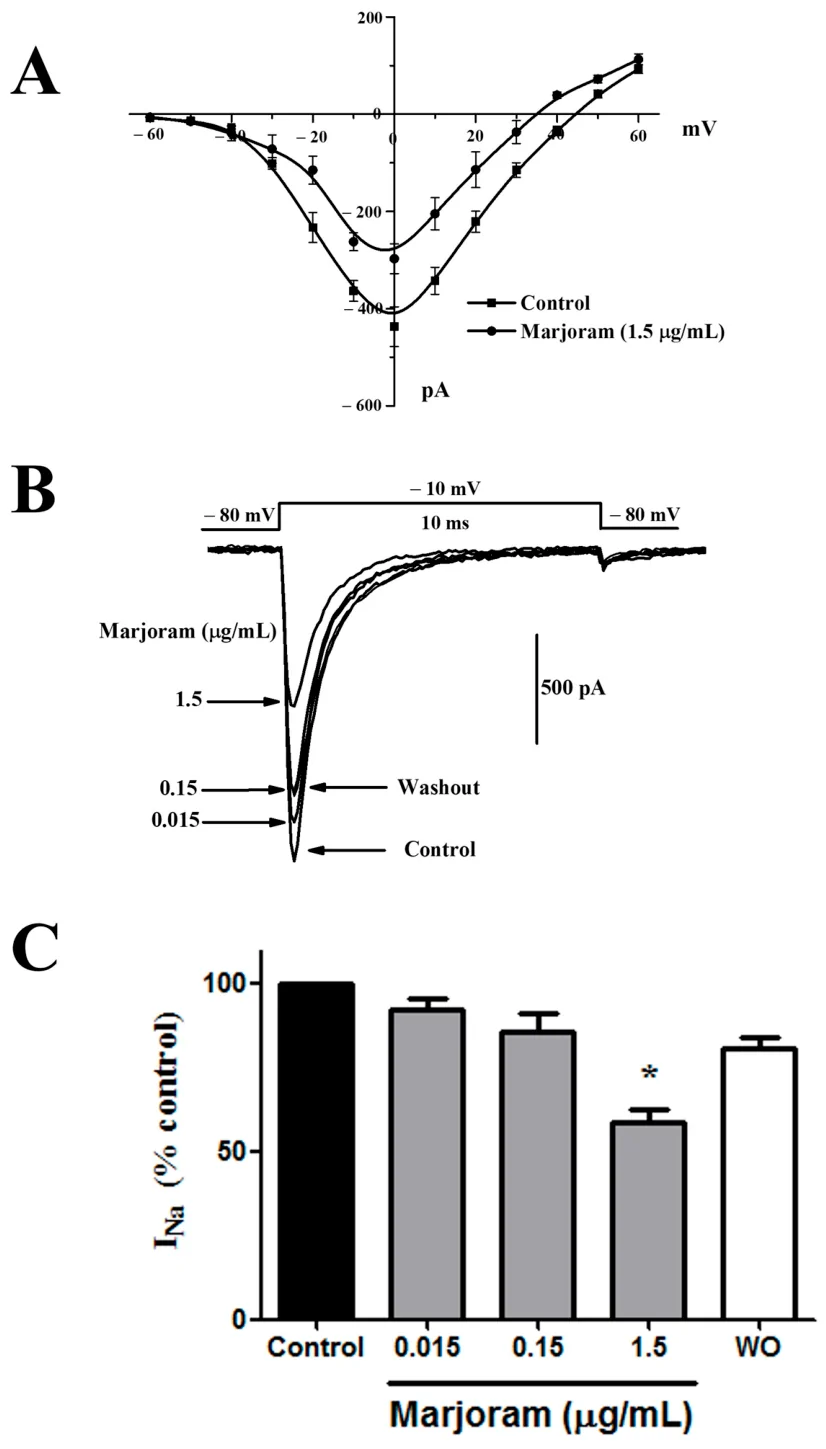
Marjoram extract partially inhibits voltage-dependent Na^+^ currents in bovine chromaffin cells. (**A**) Current–voltage relationship of whole-cell inward Na^+^ currents recorded under control conditions and after superfusion with marjoram extract. (**B**) Representative Na^+^ current traces obtained under control conditions, during application of marjoram extract at 0.015, 0.15, and 1.5 µg/mL, and after washout. Currents were evoked by 10 ms depolarizing pulses from −80 to −10 mV at 30 s intervals. (**C**) Concentration-dependent effect of marjoram extract on normalized peak I_Na_. A total of n = 9 cells from N = 4 independent cultures were analyzed. Measurements from the same culture were averaged before statistical analysis. The concentration series was analyzed by one-way repeated-measures ANOVA followed by Holm-adjusted prespecified comparisons with control: 0.015 µg/mL, *p* = 0.361; 0.15 µg/mL, *p* = 0.361; and 1.5 µg/mL, * *p* = 0.018. At 1.5 µg/mL, the mean paired difference was −37.6 percentage points (95% CI, −54.7 to −20.5; Cohen’s dz = 3.50). Here, n denotes individual cells and N denotes independent primary cultures.

We next examined whether marjoram extract modified voltage-dependent Na^+^ currents (*I*_Na_), which are essential for the generation and propagation of action potentials in excitable cells. To obtain the current–voltage (I–V) relationship shown in Figure 3A, whole-cell inward Na^+^ currents were elicited by 10 ms depolarizing pulses to the indicated test potentials. Under control conditions, the I–V relationship showed the characteristic profile of voltage-dependent Na^+^ currents, with maximal inward current around −10 mV (1329.7 ± 102.9 pA). In the presence of marjoram extract (1.5 µg/mL), the mean current amplitude at −10 mV was reduced to 762.4 ± 30.0 pA, indicating a marked reduction in voltage-dependent Na^+^ current amplitude.

For the concentration–response experiments, currents were evoked by 10 ms depolarizing pulses from −80 to −10 mV at 30 s intervals. Representative current traces obtained under control conditions, during application of increasing concentrations of marjoram extract, and after washout are shown in Figure 3B. In Figure 3C, peak INa was normalized in each cell to its initial control amplitude (100%). Marjoram extract reduced normalized INa to 92.2 ± 3.8%, 74.6 ± 5.9%, and 62.4 ± 4.2% of control at 0.015, 0.15, and 1.5 µg/mL, respectively. Because the concentrations were tested within the same cultures, the experiment was analyzed as a repeated-measures design. After averaging recordings within each of the N = 4 cultures, a one-way repeated-measures ANOVA was followed by Holm-adjusted prespecified comparisons with control. The effects at 0.015 and 0.15 µg/mL were not significant after correction (adjusted *p* = 0.361 for both comparisons), whereas inhibition at 1.5 µg/mL remained significant (adjusted *p* = 0.018; mean paired difference, −37.6 percentage points; 95% CI, −54.7 to −20.5; Cohen’s dz = 3.50).

Taken together, these results indicate that marjoram extract partially inhibits voltage-dependent Na^+^ currents in bovine chromaffin cells, with a maximal mean inhibition of approximately 38% at 1.5 µg/mL. In the culture-level analysis, statistical significance was retained only at 1.5 µg/mL. This reduction in Na^+^ current may contribute to the suppression of action-potential firing observed in the current-clamp experiments.

### 2.5. Marjoram Extract Inhibits Putative Calcium- and Voltage-Dependent Potassium Currents

We then analyzed the effect of marjoram extract on outward K^+^ currents. In bovine chromaffin cells, depolarizing protocols evoke outward K^+^ currents comprising different components, including an early Ca^2+^-activated component largely associated with large-conductance BK channels and a more sustained voltage-dependent component. The electrophysiological protocol used here, consisting of a brief depolarizing prepulse to promote Ca^2+^ entry followed by a longer depolarizing step to activate outward K^+^ currents, is based on experimental approaches previously validated in chromaffin cells. Because the specific pharmacological controls required to identify these components were not performed in the present study, we refer to them hereafter as the putative Ca^2+^-dependent and voltage-dependent K^+^ current components.

**Figure 4:**
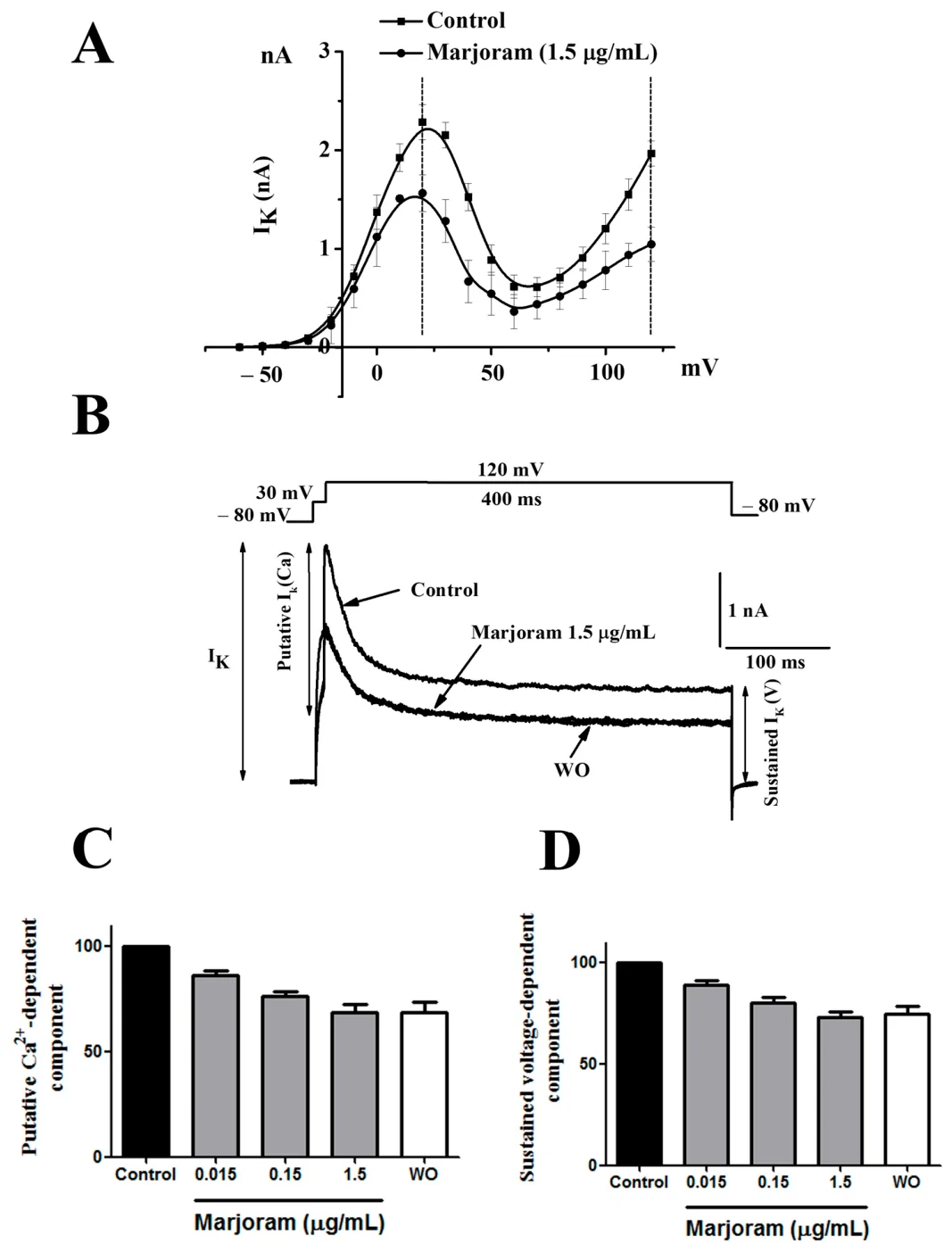
Marjoram extract inhibits putative Ca^2+^-dependent and voltage-dependent K^+^ current components. (**A**) Current–voltage relationship of outward K^+^ currents recorded in bovine chromaffin cells under control conditions and after superfusion with marjoram extract (1.5 µg/mL). (**B**) Representative K^+^ current traces obtained before and after application of marjoram extract. (**C**) Quantification of the early putative Ca^2+^-dependent component. (**D**) Quantification of the sustained voltage-dependent component. A total of n = 9 cells from N = 4 independent cultures were included in the concentration experiments. Multiple recordings from the same culture were averaged before analysis. Each concentration series was analyzed by one-way repeated-measures ANOVA. Significant overall effects were observed for the early component (F(3,9) = 11.00, *p* = 0.0023, partial η^2^ = 0.79) and the sustained component (F(3,9) = 14.90, *p* = 0.0008, partial η^2^ = 0.83). Individual concentration effects are not inferred from uncorrected pairwise tests. Here, n denotes individual cells and N denotes independent primary cultures.

As shown in Figure 4A, the current–voltage relationship revealed large outward K^+^ currents under control conditions. Superfusion with marjoram extract (1.5 µg/mL) reduced the current amplitude throughout the tested voltage range. At 20 mV, the current decreased from 2285.0 ± 279.7 pA under control conditions to 1514.5 ± 186.0 pA, whereas at 120 mV it decreased from 1968.0 ± 226.5 pA to 1546.5 ± 772.0 pA. Representative traces in Figure 4B show that marjoram extract at 1.5 µg/mL reduced both the early putative Ca^2+^-dependent component and the sustained voltage-dependent component of the outward K^+^ current.

The quantitative analysis shown in Figure 4C indicates that marjoram extract reduced the early putative Ca^2+^-dependent component to 85.9 ± 2.5%, 76.3 ± 2.1%, and 68.7 ± 3.9% of control at 0.015, 0.15, and 1.5 µg/mL, respectively. In Figure 4D, the sustained voltage-dependent component was reduced to 89.0 ± 2.2%, 80.2 ± 2.8%, and 73.2 ± 3.7% of control at the same concentrations. Because all concentrations were evaluated within the same cultures, each current component was analyzed using one-way repeated-measures ANOVA after averaging recordings within each of the N = 4 independent cultures. A significant overall treatment effect was observed for both the early putative Ca^2+^-dependent component (F(3,9) = 11.00, *p* = 0.0023, partial η^2^ = 0.79) and the sustained voltage-dependent component (F(3,9) = 14.90, *p* = 0.0008, partial η^2^ = 0.83). No uncorrected pairwise *t*-tests were used for these concentration series. Thus, the maximal mean inhibition at 1.5 µg/mL was approximately 31% for the putative Ca^2+^-dependent component and 27% for the voltage-dependent component.

Altogether, these data indicate that marjoram extract modulates not only inward Ca^2+^ and Na^+^ currents, but also outward K^+^ currents. Since K^+^ conductances are essential for membrane repolarization and action-potential termination, their inhibition may further contribute to the changes in excitability induced by marjoram extract.

### 2.6. Marjoram Extract Depolarizes the Resting Membrane Potential and Suppresses Action-Potential Firing

Because marjoram extract altered several ionic currents involved in the control of membrane excitability, we next evaluated its effects under current-clamp conditions.

**Figure 5:**
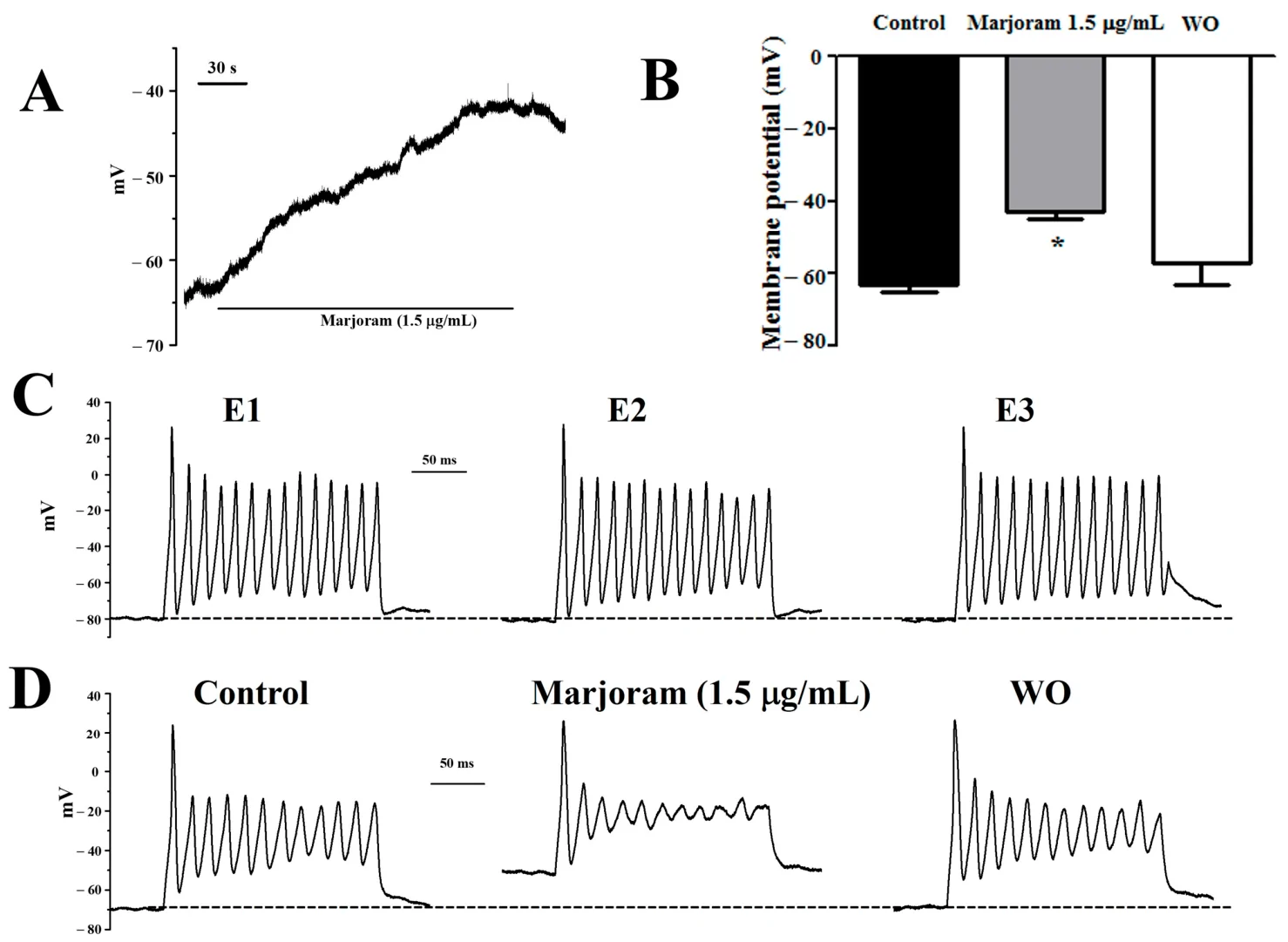
Marjoram extract depolarizes the resting membrane potential and suppresses action-potential firing. (**A**) Representative current-clamp recording showing the effect of marjoram extract (1.5 µg/mL) on the resting membrane potential of a bovine chromaffin cell. (**B**) Culture-level quantification of resting membrane potential under control conditions, during marjoram extract application, and after washout. A total of n = 5 cells from N = 3 independent cultures were recorded; values from cells derived from the same culture were averaged before analysis. The prespecified inferential contrast was paired (control vs. marjoram), whereas washout was displayed descriptively and was not included as an additional statistical comparison. Marjoram significantly depolarized the membrane potential (paired Student’s *t*-test, t(2) = 4.66, * *p* = 0.043; mean paired difference, 20.5 mV; 95% CI, 1.6 to 39.4 mV; Cohen’s dz = 2.69). (**C**) Three consecutive control responses (E1–E3) evoked by the same depolarizing current pulse (100 pA for 200 ms). (**D**) Representative recordings obtained under control conditions, during application of marjoram extract, and after washout. Panels (**C**,**D**) are representative recordings and were not treated as independent quantitative observations for inferential statistics. Here, n denotes individual cells and N denotes independent primary cultures.

As shown in Figure 5A, bovine chromaffin cells displayed a stable resting membrane potential under control conditions. Application of marjoram extract (1.5 µg/mL) produced a clear depolarization, shifting the mean membrane potential from −63.3 ± 3.3 mV under control conditions to −42.8 ± 3.5 mV during exposure to the extract (Figure 5B), corresponding to a mean depolarization of approximately 20.5 mV. The prespecified inferential comparison for resting membrane potential was the paired within-culture contrast between control and marjoram; the washout condition was included descriptively to illustrate reversibility and was not treated as an additional inferential comparison. After averaging cells within each of N = 3 independent cultures, the control-versus-marjoram difference remained significant (paired Student’s *t*-test, t(2) = 4.66, *p* = 0.043; mean paired difference, 20.5 mV; 95% CI, 1.6 to 39.4 mV; Cohen’s dz = 2.69). The membrane potential partially recovered after washout.

We next examined the ability of the cells to generate action potentials in response to depolarizing current injection. Under control conditions, repeated injections of a 100 pA current pulse for 200 ms evoked reproducible trains of action potentials (Figure 5C). In the presence of marjoram extract (1.5 µg/mL), the same stimulus markedly suppressed repetitive action-potential firing, although an initial action potential and small-amplitude membrane oscillations remained detectable (Figure 5D). Repetitive firing partially recovered after washout.

Taken together, these results indicate that marjoram extract markedly modifies the electrical excitability of bovine chromaffin cells. The depolarization of the resting membrane potential, together with the inhibition of voltage-dependent Na^+^, Ca^2+^, and K^+^ currents, is consistent with the marked suppression of repetitive action-potential firing. Since action potentials recruit voltage-dependent Ca^2+^ channels and trigger Ca^2+^-dependent secretory responses, these effects may influence stimulus–secretion coupling.

### 2.7. Marjoram Extract Differentially Modulates Relative Cytosolic Calcium Elevations Evoked by K^+^, Caffeine, and Histamine

To further investigate the effects of marjoram extract on intracellular Ca^2+^ homeostasis, relative cytosolic Ca^2+^ signals were monitored as changes in normalized Fluo-4 fluorescence in populations of bovine chromaffin cells loaded with Fluo-4-AM (10 µM). Cells were stimulated with 35 mM K^+^, caffeine (20 mM), or histamine (100 µM) to compare responses predominantly associated with voltage-dependent Ca^2+^ entry and with Ca^2+^ mobilization from ryanodine-sensitive or IP_3_-sensitive intracellular stores, respectively.

**Figure 6:**
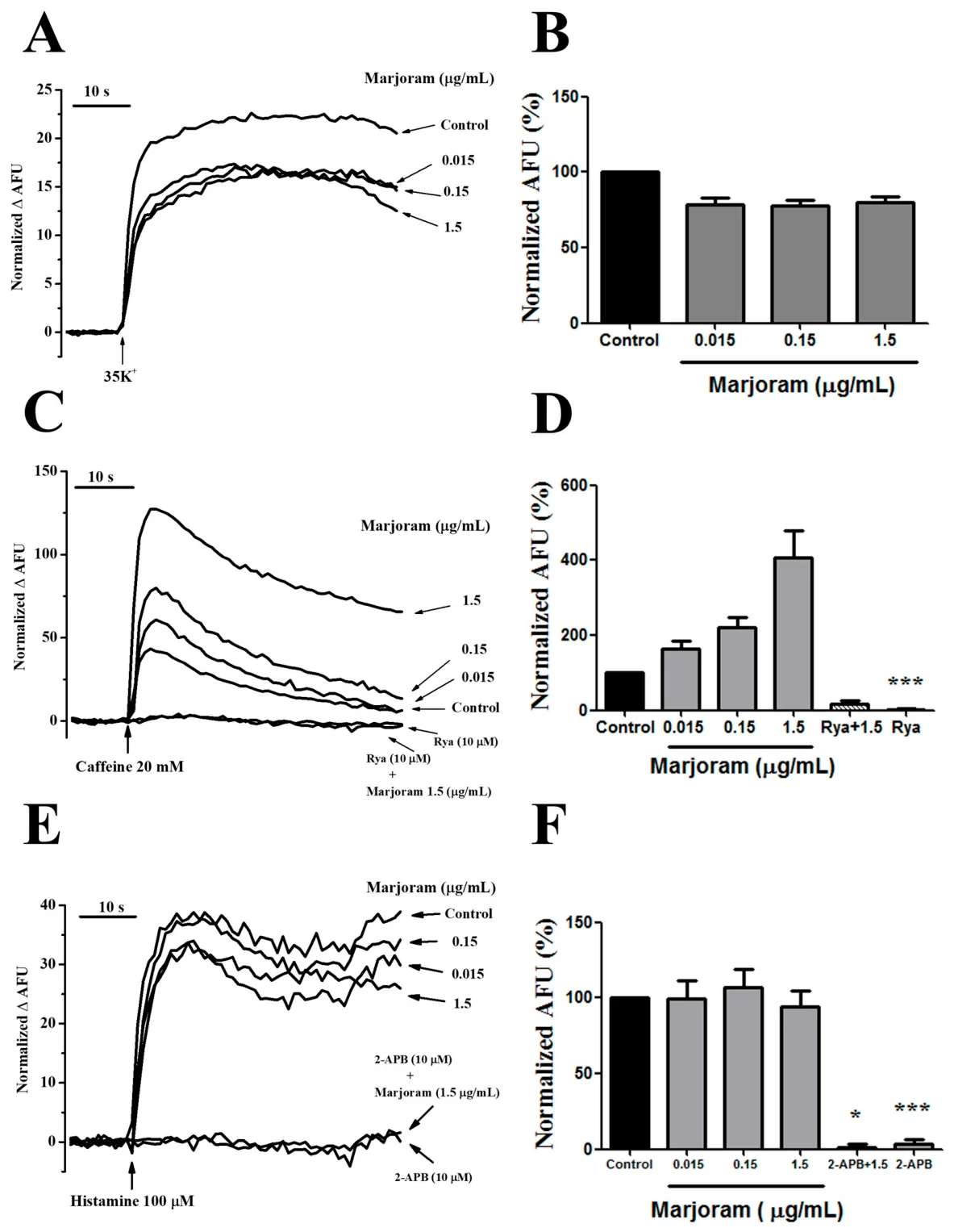
Marjoram extract differentially affects relative cytosolic Ca^2+^ signals evoked by K^+^, caffeine, and histamine. Changes in normalized Fluo-4 fluorescence were measured in populations of bovine chromaffin cells loaded with Fluo-4-AM (10 µM). (**A**) Representative responses evoked by 35 mM K^+^ after 5 min preincubation with marjoram extract. (**B**) Culture-level analysis of K^+^-evoked responses: n = 12 wells from N = 4 independent cultures; replicate wells were averaged within culture before analysis. The concentration series was analyzed by one-way repeated-measures ANOVA, and the overall effect did not reach statistical significance (F(3,9) = 2.99, *p* = 0.0883, partial η^2^ = 0.50). (**C**) Representative caffeine-evoked responses (20 mM). (**D**) Culture-level analysis of caffeine-evoked responses: n = 15 wells from N = 5 independent cultures. Extract-specific inferential comparisons were performed for 0.015, 0.15, and 1.5 µg/mL marjoram extract (final DMSO concentrations of 0.001%, 0.01%, and 0.1%, respectively) using a repeated-measures framework followed by Holm-adjusted planned comparisons with control; none was statistically significant (all adjusted *p* = 0.183). Rya denotes ryanodine. Ryanodine (Rya, 10 µM) was included as a prespecified pharmacological control and significantly reduced the caffeine-evoked response compared with control (adjusted *p* < 0.0002). The combined Rya + marjoram condition contained ryanodine (10 µM) and marjoram extract (1.5 µg/mL; final DMSO 0.1%). This combined condition is shown as a pharmacological-control condition and is interpreted descriptively; no separate inferential conclusion is drawn from this condition. The inhibitory effect of ryanodine supports the involvement of ryanodine-sensitive intracellular stores in the caffeine response but does not demonstrate direct activation or sensitization of RyR receptors by marjoram extract. (**E**) Representative histamine-evoked responses (100 µM). (**F**) Culture-level analysis of histamine-evoked responses: n = 15 wells from N = 5 independent cultures. The marjoram concentration series was analyzed by one-way repeated-measures ANOVA followed by Holm-adjusted planned contrasts; no marjoram concentration was statistically significant (all adjusted *p* > 0.05). 2-APB denotes 2-aminoethoxydiphenyl borate. 2-APB (10 µM) and 2-APB (10 µM) plus marjoram extract (1.5 µg/mL) were analyzed as separate prespecified paired pharmacological-control contrasts against control with Holm correction (adjusted *p* < 0.0002 and adjusted *p* = 0.012, respectively). In all panels, the independent culture was the biological unit for inferential statistics. Here, n denotes replicate wells and N denotes independent primary cultures. * *p* < 0.05 and *** *p* < 0.001 for the indicated Holm-adjusted pharmacological-control comparisons.

Stimulation with 35 mM K^+^ produced a reproducible increase in normalized Fluo-4 fluorescence. As shown in Figure 6A, 5 min preincubation with marjoram extract lowered the mean K^+^-evoked response. The quantitative analysis in Figure 6B showed responses of 78.6 ± 4.4%, 77.5 ± 4.4%, and 79.8 ± 3.9% of control at 0.015, 0.15, and 1.5 µg/mL, respectively. After replicate wells were averaged within each of the N = 4 independent cultures, the overall concentration effect did not reach statistical significance (repeated-measures ANOVA, F (3,9) = 2.99, *p* = 0.0883, partial η^2^ = 0.50). Thus, the approximately 20% reduction in the mean K^+^-evoked Ca^2+^ signal should be interpreted as a consistent directional trend rather than a statistically confirmed concentration effect at the culture level.

In contrast, caffeine (20 mM), which evokes Ca^2+^ mobilization from ryanodine-sensitive intracellular stores, produced larger mean responses in the presence of marjoram extract (Figure 6C). The mean responses were 157.9 ± 19.5%, 208.6 ± 25.8% and 386.9 ± 70.4% of control at 0.015, 0.15 and 1.5 µg/mL, respectively, with the largest mean response observed at 1.5 µg/mL. Accordingly, extract-specific inferential comparisons were restricted to 0.015, 0.15, and 1.5 µg/mL dry extract (final DMSO 0.001%, 0.01%, and 0.1%, respectively). These three concentrations were evaluated within culture using the repeated-measures framework described in the Methods Section, followed by Holm-adjusted planned comparisons with control; none remained statistically significant (all adjusted *p* = 0.183). Ryanodine (Rya, 10 µM) markedly reduced the caffeine-evoked response compared with control (adjusted *p* < 0.0002), supporting the involvement of ryanodine-sensitive intracellular stores in caffeine-induced Ca^2+^ mobilization. The combined pharmacological-control condition contained ryanodine (10 µM) and marjoram extract (1.5 µg/mL; final DMSO 0.1%). This combined condition is shown descriptively and was not used to support a separate inferential conclusion regarding the effect of marjoram in the presence of ryanodine. Accordingly, although the ryanodine control confirms the involvement of ryanodine-sensitive intracellular stores in the caffeine response, these experiments do not demonstrate that RyR receptors are a direct molecular target of marjoram extract or that ryanodine-sensitive stores are required for the extract-associated effect.

To determine whether the effect extended to another intracellular Ca^2+^-release pathway, cells were stimulated with histamine (100 µM), which predominantly mobilizes Ca^2+^ through IP_3_-sensitive stores in bovine chromaffin cells. Marjoram extract did not significantly modify the histamine-evoked response, which remained at 99.3 ± 12.7%, 107.1 ± 12.3%, and 94.1 ± 10.4% of control at 0.015, 0.15, and 1.5 µg/mL, respectively. The control and marjoram concentrations were analyzed as a one-way repeated-measures series at the culture level, followed by Holm-adjusted planned comparisons; none of the concentration contrasts was significant (all adjusted *p* > 0.05). The 2-APB and 2-APB plus marjoram conditions were analyzed as separate prespecified paired pharmacological-control contrasts against control with Holm correction and both reduced the histamine response (adjusted *p* < 0.0002 and *p* = 0.012, respectively).

Taken together, the culture-level analysis indicates a differential pattern of Ca^2+^ signaling responses: the K^+^-evoked and caffeine-evoked mean responses changed in opposite directions, whereas histamine-evoked responses were unchanged. Because the K^+^ and caffeine concentration effects did not reach multiplicity-adjusted significance at the culture level, these findings should be interpreted as directional associations consistent with distinct effects on plasma-membrane Ca^2+^ entry and ryanodine-sensitive intracellular stores, rather than as definitive evidence of pathway-selective modulation.

### 2.8. Effects of Marjoram Extract on MTT-Reducing Activity in Bovine Chromaffin Cells and Cortical Neurons Exposed to Veratridine or Oligomycin/Rotenone

**Figure 7:**
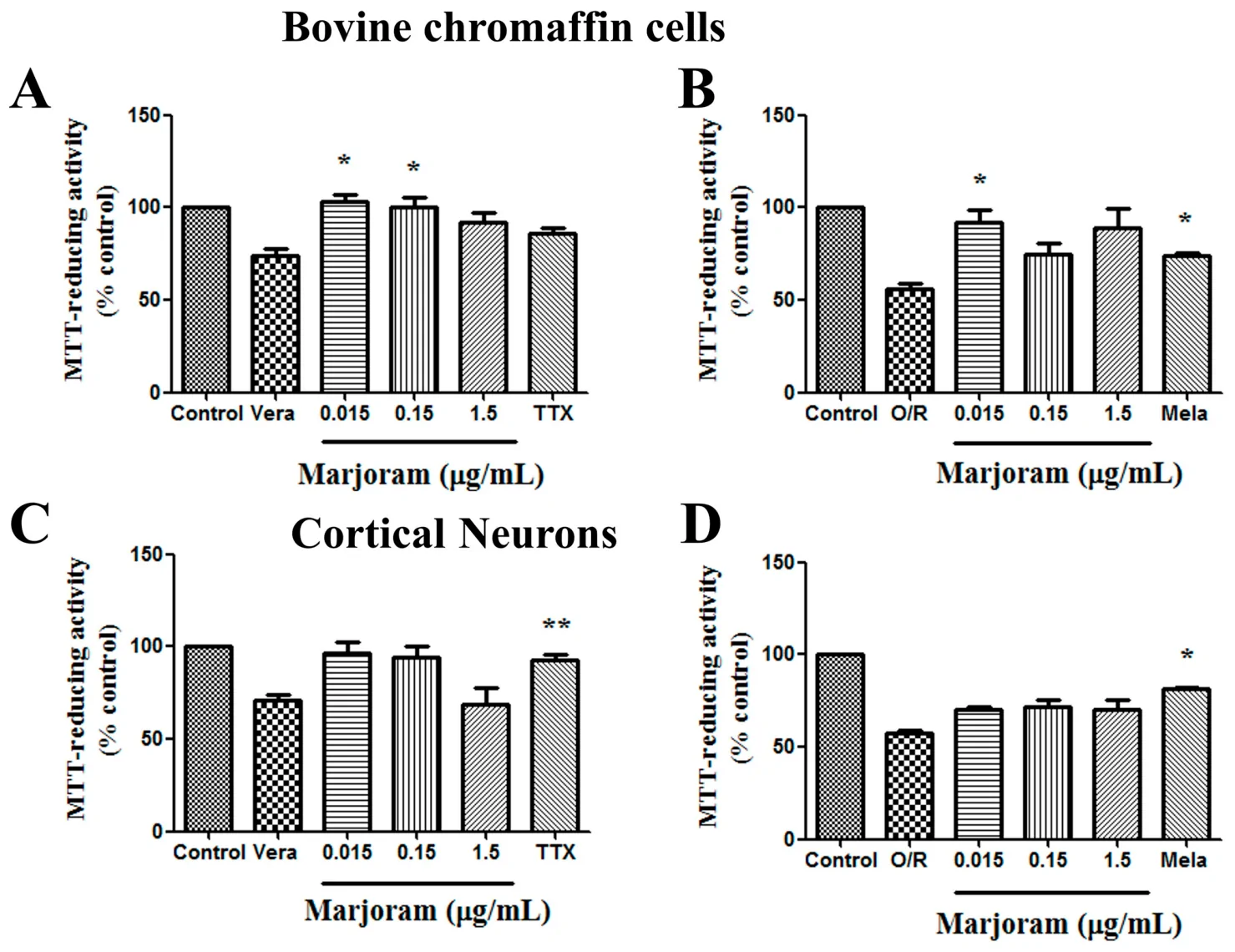
Marjoram extract and MTT-reducing activity in bovine chromaffin cells and rat cortical neurons exposed to toxic stimuli. MTT-reducing activity was measured after exposure to veratridine or oligomycin/rotenone in the absence or presence of marjoram extract. Replicate wells from the same independent culture were averaged before inferential analysis, and the independent culture was used as the biological unit. Within each panel, conditions were evaluated in the same cultures and were therefore analyzed using one-way repeated-measures ANOVA followed by prespecified comparisons against the corresponding toxic-stimulus group with Holm correction. (**A**) Bovine chromaffin cells exposed to veratridine: n = 8 wells, N = 4 cultures; adjusted *p* = 0.043, 0.034, and 0.128 for 0.015, 0.15, and 1.5 µg/mL marjoram, respectively, and *p* = 0.128 for TTX. (**B**) Bovine chromaffin cells exposed to oligomycin/rotenone (O/R): n = 5 wells, N = 4 cultures; adjusted *p* = 0.034, 0.070, and 0.067 for marjoram and *p* = 0.034 for melatonin (Mela). (**C**) Primary rat cortical neurons exposed to veratridine (Vera): n = 5 wells, N = 4 cultures; adjusted *p* = 0.102, 0.076, and 0.827 for marjoram and *p* = 0.0073 for TTX (Tetrodotoxin). (**D**) Primary rat cortical neurons exposed to oligomycin/rotenone: n = 5 wells, N = 4 cultures; adjusted *p* = 0.051, 0.104, and 0.104 for marjoram and *p* = 0.023 for melatonin. Data are presented as mean ± SEM of culture-level values. No extract-only, concentration-matched vehicle, cell-free extract/MTT interference, or orthogonal assays of cell number or membrane integrity were included; therefore, these data represent MTT-reducing activity only and should not be interpreted as evidence of cytoprotection, neuroprotection, or antioxidant activity. Here, n denotes replicate wells and N denotes independent primary cultures. * *p* < 0.05 and ** *p* < 0.01 for the indicated Holm-adjusted pharmacological-control comparisons.

Finally, we investigated whether marjoram extract preserved MTT-reducing activity under toxic conditions (see Figure 7). MTT-based assays were performed in bovine chromaffin cells and primary rat cortical neurons using two paradigms: veratridine-induced Ca^2+^ overload and oligomycin/rotenone-induced mitochondrial stress.

As shown in Figure 7A, in bovine chromaffin cells, exposure to veratridine (Vera, 30 µM) produced a marked reduction in MTT-reducing activity. Marjoram extract increased the mean MTT-reducing activity from 73.73 ± 6.06% in the veratridine-treated group to 103.10 ± 4.13%, 100.31 ± 5.66%, and 92.18 ± 6.13% at 0.015, 0.15, and 1.5 µg/mL, respectively. Replicate wells were averaged within each of the N = 4 independent cultures, and the treatment series was analyzed by one-way repeated-measures ANOVA followed by prespecified comparisons against the veratridine group with Holm correction. The effects at 0.015 and 0.15 µg/mL remained significant (adjusted *p* = 0.043 and *p* = 0.034), whereas 1.5 µg/mL did not (adjusted *p* = 0.128). Tetrodotoxin (TTX, 1 µM) yielded 86.06 ± 4.06% and did not remain significant after multiplicity correction (adjusted *p* = 0.128).

In the oligomycin/rotenone paradigm, oligomycin/rotenone (O/R) reduced MTT-reducing activity in bovine chromaffin cells to 55.60 ± 6.03% (Figure 7B). Marjoram extract yielded mean values of 91.88 ± 8.28%, 74.64 ± 9.70%, and 89.10 ± 13.40% at 0.015, 0.15, and 1.5 µg/mL, respectively. After averaging replicate wells within each of the N = 4 cultures, the repeated-measures treatment series was analyzed by one-way repeated-measures ANOVA followed by Holm-adjusted prespecified comparisons against the O/R group. A significant difference remained at 0.015 µg/mL (adjusted *p* = 0.034), whereas 0.15 and 1.5 µg/mL did not remain significant (adjusted *p* = 0.070 and *p* = 0.067, respectively). Melatonin (10 µM) yielded 74.20 ± 2.30% and remained significant after correction (adjusted *p* = 0.034).

Similar experiments were performed in primary rat cortical neurons. Veratridine reduced MTT-reducing activity to 71.08 ± 4.26% (Figure 7C). Marjoram extract yielded mean values of 96.18 ± 7.72%, 94.27 ± 7.50%, and 68.38 ± 10.20% at 0.015, 0.15, and 1.5 µg/mL, respectively. Culture-level values (N = 4) were analyzed as a one-way repeated-measures treatment series followed by Holm-adjusted prespecified comparisons against the veratridine group. None of the marjoram comparisons remained statistically significant (adjusted *p* = 0.102, 0.076, and 0.827, respectively). TTX yielded 92.42 ± 3.93% and remained significant (adjusted *p* = 0.0073).

Likewise, as shown in Figure 7D, in the oligomycin/rotenone paradigm, O/R reduced MTT-reducing activity in cortical neurons to 57.21 ± 3.40%. Marjoram extract yielded mean values of 70.31 ± 2.49%, 71.73 ± 7.20%, and 70.05 ± 8.30% at 0.015, 0.15, and 1.5 µg/mL, respectively. Culture-level values (N = 4) were analyzed by one-way repeated-measures ANOVA followed by Holm-adjusted prespecified comparisons against the O/R group. None of the marjoram comparisons reached the multiplicity-adjusted significance threshold (adjusted *p* = 0.051, 0.104, and 0.104, respectively). Melatonin yielded 81.27 ± 1.25% and remained significant (adjusted *p* = 0.023).

Overall, the culture-level analysis identified statistically significant preservation of MTT-reducing activity in selected bovine chromaffin-cell conditions, particularly at the lower marjoram concentrations, whereas the corresponding marjoram comparisons in cortical neurons did not remain statistically significant after correction for multiple comparisons. These findings are therefore restricted to the MTT endpoint and should not be interpreted as evidence that marjoram preserved cell number, membrane integrity, or survival.

The MTT assay reflects cellular metabolic reducing capacity and does not directly measure cell number, membrane integrity, apoptosis, or necrosis. In addition, the original experimental series did not include extract-only groups, concentration-matched vehicle controls for each extract condition, cell-free wells containing marjoram extract plus MTT reagents, a dedicated assessment of absorbance interference at 540 nm, or an independent orthogonal assay of cell number or membrane integrity. Consequently, direct chemical reduction of MTT, optical interference, or extract-induced changes in cellular metabolism cannot be fully distinguished from changes in cell number or membrane integrity. The present results are therefore described exclusively as preservation of MTT-reducing activity. Confirmation with complementary assays such as LDH release, resazurin, calcein/propidium iodide, Annexin V, or related approaches would be required before drawing conclusions regarding cytoprotection or neuroprotection.

**Figure 8:**
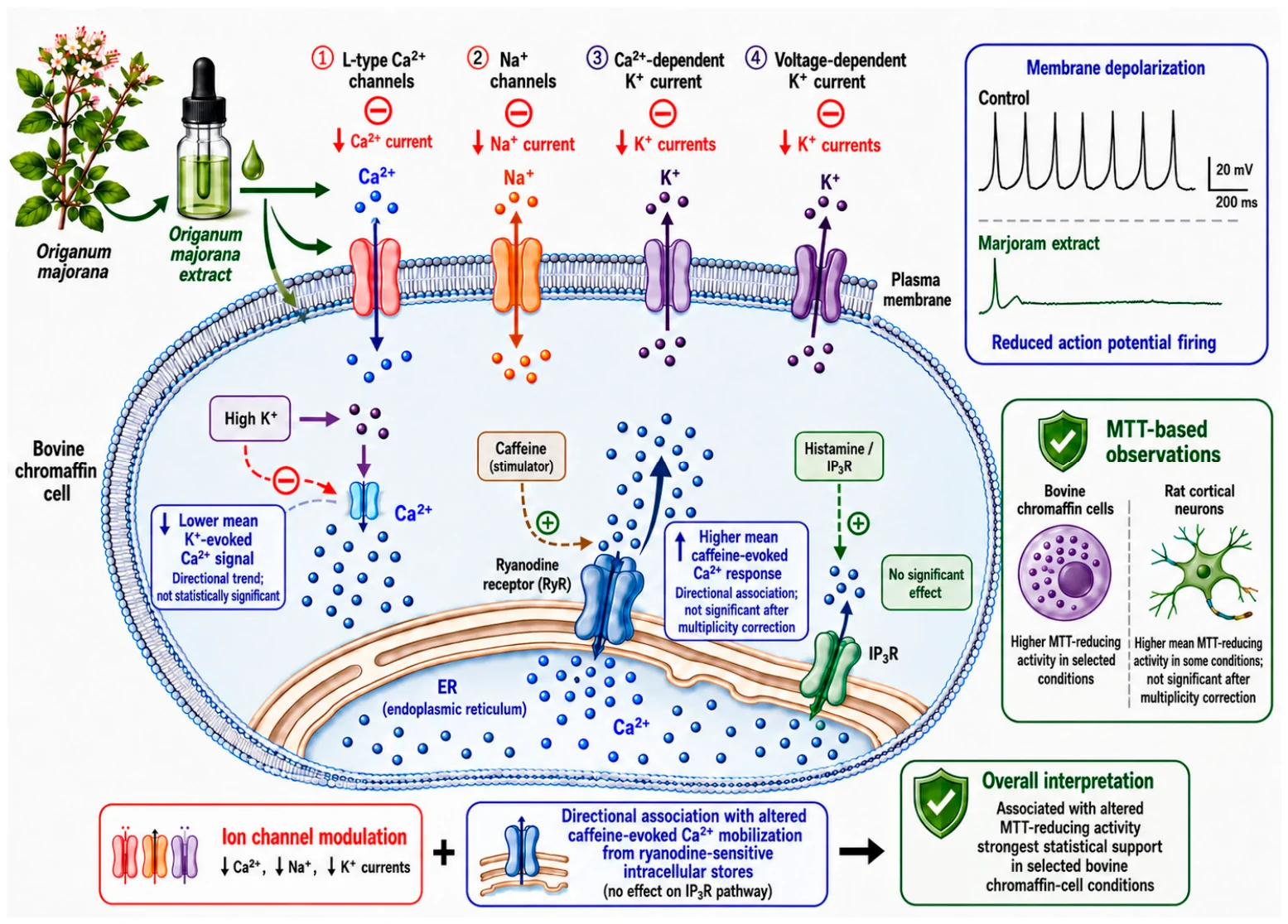
Proposed working model of marjoram extract effects on excitability, Ca^2+^ handling, and MTT-reducing activity. Schematic summary integrating the electrophysiological, Ca^2+^-imaging, and MTT-based findings. The ion-current effects are supported by culture-level statistical analysis, whereas the K^+^-evoked and caffeine-evoked Ca^2+^ changes should be interpreted as directional associations because the concentration effects did not remain significant after culture-level analysis and multiplicity correction. Likewise, MTT-reducing activity was preserved significantly only in selected conditions. The scheme therefore represents a hypothesis-generating integration of the data and does not establish direct RyR activation, antioxidant activity, cytoprotection, or neuroprotection. Any depiction of RyR in the scheme denotes the ryanodine-sensitive intracellular store pathway and should not be interpreted as evidence that the receptor is a demonstrated direct molecular target of the extract.

Figure 8 provides a schematic summary of the cellular actions of *Origanum majorana* extract derived from the present results. Marjoram appears to exert a multimodal modulation of chromaffin cell excitability by acting on several plasma-membrane ion currents. At the membrane level, the extract partially inhibits L-type Ca^2+^ currents, Na^+^ currents, and both putative Ca^2+^-dependent and voltage-dependent K^+^ currents. These combined effects are consistent with depolarization of the resting membrane potential and a marked suppression of repetitive action-potential firing, indicating a profound alteration of the electrical behavior of bovine chromaffin cells.

In parallel, the mean Ca^2+^-imaging responses showed a differential pattern. K^+^-evoked relative cytosolic Ca^2+^ signals were lower in the presence of marjoram, whereas caffeine-evoked responses were higher and histamine-evoked responses were essentially unchanged. However, after the culture-level analysis and correction for multiple comparisons, the K^+^ and caffeine concentration effects did not reach statistical significance. Accordingly, Figure 8 should be interpreted as a working model summarizing directional associations that are compatible with differential regulation of plasma-membrane Ca^2+^ entry and caffeine/ryanodine-sensitive stores, rather than as a definitive mechanistic scheme.

The MTT data likewise showed preservation of MTT-reducing activity only in selected conditions, with the most consistent statistically supported effects observed in bovine chromaffin cells at lower marjoram concentrations. Overall, the figure integrates the electrophysiological, Ca^2+^-imaging, and MTT-based observations into a hypothesis-generating model of ion-current modulation, Ca^2+^ handling, and MTT-reducing activity rather than a causal model of cytoprotection or neuroprotection.

## 3. Discussion

In the present study, we provide evidence that *Origanum majorana* extract modulates bovine chromaffin-cell excitability and Ca^2+^ homeostasis and is associated with preservation of MTT-reducing activity under selected toxic conditions. The principal electrophysiological findings were partial inhibition of voltage-dependent Ca^2+^ and Na^+^ currents, modulation of putative Ca^2+^-dependent and voltage-dependent K^+^ current components, membrane depolarization, and marked suppression of repetitive action-potential firing. In parallel, mean K^+^-evoked Fluo-4 responses were lower and mean caffeine-evoked responses were higher, whereas histamine-evoked responses were essentially unchanged. However, the K^+^ and caffeine concentration effects did not remain statistically significant after culture-level analysis and multiplicity correction. MTT-based findings are likewise interpreted as changes in MTT-reducing activity rather than direct evidence of cytoprotection or neuroprotection.

### 3.1. Modulation of Membrane Ion Channels

The first relevant electrophysiological observation was the partial inhibition of voltage-dependent Ca^2+^ currents by marjoram extract. The maximal reduction was approximately 20% of the total Ca^2+^ current and was not additive with nifedipine, a dihydropyridine blocker of L-type channels. This pharmacological profile is consistent with preferential inhibition of the L-type component rather than with nonspecific blockade of all voltage-dependent Ca^2+^ channel subtypes. Bovine chromaffin cells express several Ca^2+^ channel populations, and L-type channels contribute to subthreshold Ca^2+^ entry, pacemaker-like activity and sustained Ca^2+^-dependent responses, whereas N- and P/Q-type channels are more closely linked to fast exocytosis [13,14,15,16,21,22]. Thus, the magnitude and pharmacological characteristics of the effect observed here are consistent with preferential inhibition of L-type-dependent Ca^2+^ entry without complete suppression of global Ca^2+^ influx. This interpretation is also consistent with the approximately 20% decrease in K^+^-evoked cytosolic Ca^2+^ signals observed in the imaging experiments. The lack of a further reduction when nifedipine and marjoram were combined, irrespective of the order of application, is particularly important because it argues against a simple additive blockade of independent Ca^2+^-channel populations. Although this does not demonstrate a direct interaction of any extract constituent with L-type channel proteins, it supports the interpretation that the L-type component is preferentially affected by the extract.

The Ca^2+^ channel inhibitory profile is also compatible with previous observations in non-neuronal preparations. Marjoram extracts have been reported to relax intestinal smooth muscle and to produce verapamil-like effects, suggesting inhibition of voltage-dependent Ca^2+^ entry as one component of their antispasmodic and antidiarrheal actions [3,12]. The present results extend this type of membrane action to an excitable neuroendocrine model, although the molecular determinants of the effect remain to be established.

Marjoram extract also partially inhibited voltage-dependent Na^+^ currents. Because Na^+^ channels are essential for the rapid depolarizing phase and repetitive generation of action potentials, this effect provides a plausible contribution to the marked suppression of evoked firing observed under current-clamp conditions. A comparable electrophysiological relationship between combined changes in Na^+^ and Ca^2+^ currents and altered action-potential generation has been described previously in bovine chromaffin cells [23]. In the present study, inhibition of Na^+^ conductance occurred together with changes in Ca^2+^ and K^+^ currents, indicating that the extract acts on several components that collectively determine membrane excitability.

The extract reduced both the early putative Ca^2+^-dependent and the sustained voltage-dependent components of the outward K^+^ current. The decrease in the putative Ca^2+^-dependent component may partly result from reduced Ca^2+^ entry, since activation of BK channels in chromaffin cells is tightly coupled to local Ca^2+^ influx through voltage-dependent Ca^2+^ channels [24,25,26]. Because specific pharmacological separation of the K^+^-current components was not performed in the present study, this interpretation remains functional rather than molecular. The inhibition of the sustained voltage-dependent component further suggests additional effects on membrane conductance. Consistent with these combined actions, marjoram depolarized the resting membrane potential and almost abolished evoked action-potential firing. Sustained depolarization may itself reduce Na^+^ channel availability through voltage-dependent inactivation, while inhibition of Ca^2+^ and K^+^ conductances would alter both depolarizing and repolarizing phases. Overall, the electrophysiological data indicate a broad modulation of excitability rather than an effect restricted to a single membrane channel. This integrated effect is relevant because chromaffin-cell firing depends on the coordinated activity of Na^+^, Ca^2+^ and K^+^ channels rather than on any single conductance [15,27]. The partial reversibility observed after washout also suggests that the acute electrophysiological actions cannot be interpreted simply as irreversible loss of cell function.

### 3.2. Intracellular Ca^2+^ Responses

A particularly relevant observation was the differential direction of the Ca^2+^-imaging responses. In agreement with the electrophysiological data, the mean normalized Fluo-4 response evoked by high K^+^ was lower in the presence of marjoram extract, whereas mean caffeine-evoked responses were higher and the histamine-induced response remained essentially unchanged. After culture-level analysis and multiplicity correction, however, the K^+^ and caffeine concentration effects did not reach statistical significance. Caffeine-sensitive and IP_3_-sensitive stores represent functionally distinguishable Ca^2+^-release pathways in bovine chromaffin cells [28,29,30,31]. Taken together, the present pattern is compatible with, but does not demonstrate, differential behavior of ryanodine-sensitive and IP_3_-sensitive intracellular Ca^2+^ stores.

The apparently opposite changes in plasma-membrane Ca^2+^ entry and caffeine-evoked intracellular release are not necessarily contradictory because they involve different components of Ca^2+^ homeostasis. The higher mean caffeine-evoked response could reflect changes in store filling, luminal Ca^2+^ handling, or the coupling between caffeine stimulation and Ca^2+^ release from ryanodine-sensitive intracellular stores, rather than a direct effect on the RyR protein itself. Importantly, the present experiments do not demonstrate that marjoram directly activates or sensitizes RyR receptors, nor do they establish that ryanodine-sensitive intracellular stores are required for the preservation of MTT-reducing activity. The inhibitory effect of ryanodine on the caffeine response supports the involvement of ryanodine-sensitive stores in the caffeine-evoked signal, but does not identify RyRs as a molecular target of the extract. Establishing a causal role for these stores would require dedicated experiments testing the extract in the presence and absence of an RyR inhibitor under matched vehicle conditions, both for caffeine-evoked Ca^2+^ mobilization and, separately, for the MTT-reducing activity endpoint. Such experiments were not performed in the present study; therefore, the relationship remains correlative and alternative mechanisms, including altered endoplasmic-reticulum Ca^2+^ loading or release coupling, cannot be excluded.

### 3.3. MTT-Reducing Activity Under Toxic Conditions

Marjoram extract was associated with higher MTT-reducing activity in selected conditions during veratridine exposure. Veratridine produces persistent activation of voltage-dependent Na^+^ channels, sustained depolarization and secondary Ca^2+^ loading. The partial inhibition of Na^+^ currents and reduction in voltage-dependent Ca^2+^ entry observed electrophysiologically provide a plausible context for the MTT findings. However, the present experiments do not establish that these electrophysiological effects caused preservation of cell number or membrane integrity. TTX was included as a pharmacological reference for the Na^+^ channel-dependent component of the veratridine paradigm, but the MTT endpoint alone cannot distinguish preservation of cell number or membrane integrity from altered cellular reducing activity. Accordingly, the relationship between membrane-current modulation and the MTT response is described as an association rather than a protective mechanism.

Oligomycin/rotenone also reduced MTT-reducing activity, consistent with the strong dependence of tetrazolium reduction on cellular metabolic and mitochondrial function. Marjoram was associated with higher MTT-reducing activity in selected bovine chromaffin-cell conditions, but these data cannot establish an antioxidant effect because reactive oxygen species, oxidative damage biomarkers, mitochondrial membrane potential, or other direct indices of oxidative stress were not measured. Moreover, oligomycin/rotenone can directly alter the metabolic processes that contribute to MTT reduction. The present findings should therefore be interpreted only as changes in MTT-reducing activity during oligomycin/rotenone exposure, not as evidence that the extract prevented oxidative stress or mitochondrial injury.

In primary cortical neurons, the mean MTT-reducing activity was higher at 0.015 and 0.15 µg/mL marjoram in both toxic paradigms, but none of the marjoram comparisons remained statistically significant after culture-level analysis and correction for multiple comparisons. The highest concentration likewise showed no statistically supported preservation of MTT-reducing activity. These neuronal observations therefore do not provide evidence beyond the MTT-reducing activity endpoint. Differences among concentrations could reflect biological variability, metabolic modulation, assay interference, or true differences in cellular responses, and the present experimental design cannot distinguish among these possibilities.

Previous studies have reported antioxidant, anti-inflammatory and neuroprotective effects of other *O. majorana* preparations in experimental models [6,9], and clinical pilot studies have reported changes in oxidative-stress biomarkers after marjoram tea consumption [10,11]. Additional evidence of biological activity under cellular stress has been reported in an in vivo model of isoproterenol-induced cardiotoxicity, in which *O. majorana* extract exerted cardioprotective effects [32]. These reports provide biological context but cannot validate the interpretation of the present MTT assay, because the preparation, experimental systems and endpoints differ. In the present study, no direct oxidative-stress biomarker and no orthogonal assay of cell number, membrane integrity, apoptosis, or necrosis was performed. The MTT findings are therefore deliberately restricted to preservation of MTT-reducing activity.

### 3.4. Possible Compounds Contributing to the Observed Effects

The hydroalcoholic extract used in this study was characterized as a phenolic-rich preparation. Thirty-seven phenolic compounds were detected, 18 of them tentatively identified, with flavones and rosmarinic acid derivatives predominating. Among the most abundant constituents were 6-hydroxyluteolin-7-*O*-glucoside, rosmarinic acid, lithospermic acid isomer I, luteolin-O-glucoside and luteolin-7-*O*-glucoside. Phenolic compounds from *O. majorana* have previously been associated with antioxidant and cytoprotective activities [7,8]. Therefore, the preservation of MTT-reducing activity observed in selected conditions is compatible with biological activities previously reported for phenolic-rich preparations, but the present experiments do not establish that any individual compound is responsible for the electrophysiological, Ca^2+^-signaling or MTT-related effects. Because these compounds coexist at substantially different concentrations, the activity of the complete extract may also depend on interactions among phenolic acids, flavones and their glycosylated derivatives rather than on the most abundant constituent alone. This point is particularly relevant when comparing the present results with studies using purified phytochemicals.

It is also important to distinguish the hydroalcoholic preparation examined here from *O. majorana* essential oil. The analytical characterization focused on phenolic compounds, and volatile monoterpenes characteristic of essential oils were not identified or quantified in the extract. Consequently, terpinen-4-ol cannot be considered a demonstrated contributor to the present effects. Evidence concerning terpinen-4-ol derives mainly from essential-oil studies and cannot establish its involvement here. For the present extract, interpretation should remain centered on the characterized phenolic profile; assessment of volatile constituents would require dedicated GC-MS analysis and testing of purified compounds.

### 3.5. Limitations and Future Perspectives

Several limitations should be considered when interpreting these findings. First, the marjoram extract is a complex mixture, and the active compound or combination of compounds responsible for the observed effects was not identified. Fractionation of the extract and experiments with isolated major phenolics will be required to establish structure-activity relationships and determine whether membrane-channel modulation, intracellular Ca^2+^ responses and MTT-reducing activity arise from the same or different constituents. In particular, experiments combining fractionation with electrophysiology and Ca^2+^ imaging would help determine whether the same fraction reproduces both membrane-channel and intracellular-store effects. This would provide a more direct route to identifying the constituent classes responsible for each cellular action.

Second, the higher mean caffeine-evoked Ca^2+^ responses are compatible with altered mobilization from ryanodine-sensitive intracellular stores, but the concentration effect did not remain statistically significant after culture-level analysis. Ryanodine markedly reduced the caffeine-evoked response, supporting the involvement of ryanodine-sensitive intracellular stores in the caffeine response. However, the present experiments do not establish that marjoram directly activates or sensitizes RyR receptors, nor do they demonstrate that these stores are required for the extract-associated effect. Establishing a causal role would require dedicated experiments specifically designed to compare marjoram extract in the presence and absence of RyR inhibition under appropriately matched experimental conditions. No experiment tested whether RyR inhibition alters the MTT-reducing activity associated with marjoram. Thus, direct activation or sensitization of RyR receptors and a causal role of ryanodine-sensitive intracellular stores in the MTT-related effects remain unproven.

Third, MTT was the sole endpoint used to assess the response of cells to the toxic paradigms. The assay measures tetrazolium-reducing metabolic activity and does not directly quantify cell number, membrane integrity, apoptosis, or necrosis. The original experimental design did not include extract-only groups, matched vehicle-only groups for every extract concentration, cell-free wells containing extract plus MTT reagents, a dedicated test of absorbance interference at 540 nm, or an orthogonal assay of cell number, membrane integrity, apoptosis, or necrosis. Therefore, direct chemical reduction of MTT, optical interference by extract constituents, and extract-induced changes in cellular metabolism cannot be fully excluded. For this reason, the manuscript consistently reports these findings as preservation of MTT-reducing activity and does not use them as direct evidence of cytoprotection, neuroprotection, or antioxidant activity. Complementary assays such as LDH release, resazurin, calcein/propidium iodide, Annexin V, or related approaches would be required to determine whether the observed MTT changes are accompanied by preservation of cell number or membrane integrity. To address the non-independence of multiple wells obtained from the same preparation, replicate wells were averaged within each experimental condition and the independent culture was used as the biological experimental unit. Nevertheless, the relatively small number of independent cultures in these experiments limits statistical precision and should be considered when interpreting the magnitude and uncertainty of the effects.

Fourth, concurrent vehicle-only controls matched to each final DMSO concentration were not included in the original experimental series. This limitation is especially relevant to the caffeine experiment at 15 µg/mL dry extract, which contained 1% DMSO. In the absence of a matched 1% DMSO control, the response observed under this condition cannot be attributed specifically to the extract. Accordingly, the 15 µg/mL point and the ryanodine plus 15 µg/mL marjoram condition are retained only as descriptive observations and are not used for extract-specific inferential or mechanistic conclusions. The lower marjoram concentrations contained 0.001–0.1% DMSO; although the potential contribution of vehicle is smaller at these concentrations, the absence of concurrent matched vehicle controls remains a limitation of the original design. Future experiments should include a DMSO-matched vehicle control for every extract concentration, including a dedicated 1% DMSO control whenever the 15 µg/mL dry-extract concentration is tested.

Finally, most mechanistic electrophysiological and Ca^2+^-signaling experiments were performed in bovine adrenal chromaffin cells, while the cortical-neuron experiments were mainly limited to metabolic activity under toxic conditions. Chromaffin cells are a well-established excitable neuroendocrine model, but their responses cannot be assumed to reproduce those of central neurons. Accordingly, the present findings should not be directly extrapolated to neurodegenerative disease, therapeutic efficacy or clinical use. Future work should extend the electrophysiological and Ca^2+^-signaling analysis to neuronal models, evaluate the major phenolic constituents individually and in defined combinations, directly test the contribution of ryanodine-sensitive intracellular stores by combining the extract with an RyR inhibitor under matched vehicle conditions, and subsequently examine the most relevant mechanisms in disease-related and in vivo models. Such studies would also allow the concentration range producing potentially beneficial effects to be defined more precisely and would help determine whether the actions observed in chromaffin cells are preserved in neuronal preparations.

## 4. Materials and Methods

### 4.1. Plant Material and Pressurized Liquid Extraction (PLE) Conditions

Dried commercial leaves of *Origanum majorana* L., originating from Egypt, were purchased from a specialized herbal supplier (Murciana de Herboristera, Murcia, Spain). The plant material was supplied and commercially labeled as *O. majorana* L. Because it was acquired as an already processed commercial herbal product, no independent taxonomic authentication was performed by the authors and no voucher specimen was deposited in a herbarium. In addition, the commercial batch/lot number of the plant material was not retained in the archived experimental records and can no longer be reliably retrieved. The dried leaves were ground using a Grindomix GM 200 mill (Retsch, Asturias, Spain) and sieved (BA200N, CISA, Barcelona, Spain) to obtain a particle size < 500 µm. The ground plant material was stored at −20 °C until extraction.

An ASE 350 system from Dionex Corporation (Sunnyvale, CA, USA), equipped with a solvent controller unit, was used to obtain the hydroalcoholic extract. A marjoram sample (1 g) was homogeneously mixed with 4 g of sea sand and transferred to an 11 mL extraction cell. A single static extraction cycle was performed using ethanol:water (70:30, *v*/*v*) as the extraction solvent at 102.07 atm and 100 °C for 10 min. These extraction conditions had previously been optimized for phenolic compound recovery and were reported to provide an extraction yield of 15.6 ± 0.3% in an earlier study [33]. However, the extraction yield was not specifically recorded for the batch of marjoram extract used in the present biological experiments; therefore, a batch-specific extraction yield cannot be reported. The hydroalcoholic extract was recovered in a glass vial, ethanol was removed by vacuum rotary evaporation (RV 10 control VWR, IKA, Staufen, Germany), and the remaining extract was freeze-dried. The solid extract was stored at 4 °C in the dark until use.

### 4.2. Phenolic Compound Determination of Marjoram Extract by HPLC-PAD Analysis

Phenolic compounds in the marjoram extract were analyzed by HPLC-PAD according to the methodology previously described by Villalva et al. [17]. An Agilent 1260 HPLC Infinity Series system (Agilent Technologies, Santa Clara, CA, USA) coupled to a photodiode-array detector (PAD; G4212A, Agilent Technologies) was used. Chromatographic separation was performed using an ACE 3 C18-AR guard column (10 mm × 3 mm) followed by an Excell 3 Super C18 analytical column (150 mm × 4.6 mm, 3 µm particle size), maintained at 35 °C.

The mobile phase consisted of Milli-Q water containing 0.1% (*v*/*v*) formic acid (solvent A) and acetonitrile (solvent B), at a flow rate of 0.5 mL/min. The following gradient was applied: 100% A at 0 min; 100% A at 1 min; 85% A at 6 min; 75% A at 21 min; 65% A at 26 min; 50% A at 36 min; 50% A at 41 min; 0% A at 44 min; and 100% A at 49 min.

Freeze-dried marjoram extract was dissolved in DMSO at 1.5 mg/mL, filtered through a 0.45 µm PVDF membrane filter (Symta, Madrid, Spain), and a 20 µL aliquot was injected into the HPLC system. PAD signals were recorded at 280 nm for phenolic acids, 320 nm for hydroxycinnamic acids, 340 nm for flavones, and 360 nm for flavonols. Individual phenolic compounds were identified on the basis of retention time, UV–Vis spectral characteristics, MS/MS fragmentation patterns, and comparison with authentic standards when available, according to previously established criteria [17,18]. Ethyl gallate was used as an internal standard.

Phenolic compounds were quantified using calibration curves generated with authentic standards or, when an authentic standard was unavailable, with the closest structurally related standard. Specifically, gallic acid was used for 3,4-dihydroxyphenyllactic acid; caffeic acid for caffeic acid hexoside; orientin for luteolin-C-hexoside; luteolin-7-*O*-glucoside for 6-hydroxyluteolin-7-*O*-glucoside, luteolin rutinoside, and luteolin-O-glucoside; lithospermic acid for lithospermic acid isomers and the sagecoumarin isomer; salvianolic acid B for salvianolic acid isomers; rosmarinic acid for rosmarinic acid derivatives; and apigenin for hydroxymethoxy flavones. Results were expressed as mg/g dry extract and represent the mean of three analytical replicates.

For all biological experiments, the freeze-dried marjoram extract was dissolved in DMSO at a stock concentration of 1.5 mg/mL (1.5 µg/µL). Extract concentrations are reported throughout the manuscript primarily as dry-extract mass concentrations (µg/mL), with the corresponding stock-volume equivalents (µL/mL) provided in parentheses where appropriate. The experimental concentrations included in the analyses were 0.015 µg/mL (0.01 µL/mL), 0.15 µg/mL (0.1 µL/mL), and 1.5 µg/mL (1 µL/mL), corresponding to final DMSO concentrations of 0.001%, 0.01%, and 0.1% (*v*/*v*), respectively.

### 4.3. Isolation and Culture of Bovine Chromaffin Cells

The animal study protocol was approved by the local Animal Care Committee of Universidad Autónoma de Madrid (approval code ES280790000092) and authorized by the Dirección General de Agricultura, Ganadería y Alimentación, Consejería de Medio Ambiente, Agricultura e Interior, Comunidad de Madrid (project reference PROEX 221.6/24; authorization dated 19 August 2024). All experiments were conducted in accordance with the ethical principles and guidelines established by Directive 2010/63/EU on the protection of animals used for scientific purposes and Spanish Royal Decree 53/2013.

Chromaffin cells, as sympathetic neurons, are developed from the neural crest. They are excitable cells with neuron-like electrical properties [27,34] with the capacity to synthesize, store and release adrenaline and noradrenaline (for review, see ref. [35]). They are one of the most popular and widely used cellular models for investigating the molecular mechanisms underlying cellular excitability and neurotransmitter release [15,16,36,37].

In accordance with bioethical animal welfare practices and European regulations (EC No. 1099/2009), Spanish legislation requires a procedure that avoids as much pain and agony of the animal until its death. Adrenal glands were obtained from a local slaughterhouse under the supervision of the local veterinary service. For the stunning and slaughter of the animal, a punch gun actuated by a captive bullet cartridge is used. The end of the barrel is attached to the animal’s skull and fired. Bleeding by cutting the skin with a knife begins immediately after stunning. Bovine chromaffin cells were isolated by digestion of the adrenal medulla with collagenase. A total of 24 adrenal glands from 12 animals were distributed across 12 independent cell cultures. The figure captions indicate the number of cells used and the number of cultures used for each group of experiments. For each primary culture, two adrenal glands were pooled before cell plating. Briefly, the isolated cells were suspended in Dulbecco’s modified Eagle’s medium (DMEM) supplemented with 5% fetal bovine serum, 50 IU/mL penicillin, and 50 µg/mL streptomycin. To limit fibroblast proliferation, the culture medium was additionally supplemented with the proliferation inhibitors cytosine arabinoside (10 µM), fluorodeoxyuridine (10 µM), and leucine methyl ester (10 µM). For patch-clamp studies, cells were plated on 1 cm-diameter glass coverslips at low density (5 × 10^4^ cells per coverslip). For intracellular Ca^2+^ and MTT measurements, bovine chromaffin cells were seeded at 2 × 10^5^ cells per well into 96-well plates. Cultures were maintained in an incubator at 37 °C in a water-saturated environment with 5% CO_2_. Cells were used 1–4 days after plating.

### 4.4. Isolation and Culture of Cortical Neurons

All experiments were conducted in accordance with the ethical principles and guidelines established by Directive 2010/63/EU on the protection of animals used for scientific purposes and Spanish Royal Decree 53/2013. The animal study protocol was approved by the local Animal Care Committee of Universidad Autónoma de Madrid (approval code ES280790000092) and authorized by the Dirección General de Agricultura, Ganadería y Alimentación, Consejería de Medio Ambiente, Agricultura e Interior, Comunidad de Madrid (project reference PROEX 221.6/24; authorization dated 19 August 2024). All animals used in this study were provided by the Animal Facility of the Faculty of Medicine, Universidad Autónoma de Madrid. Every effort was made to minimize the number of animals used and their suffering.

Primary cortical neurons were prepared from the brains of embryonic day 18 (E18) Sprague-Dawley rat embryos obtained from pregnant rats weighing 250–300 g, as previously described [38]. Four pregnant rats were used to obtain approximately 40–45 E18 embryos, from which four independent primary cortical neuronal cultures (N = 4) were prepared. Embryos obtained from each pregnant rat were processed according to the same standardized culture procedure. Pregnant rats were sacrificed by decapitation under sodium pentobarbital anesthesia (60 mg/kg, i.p.), and the embryonic heads were immediately placed in 1× Locke’s solution containing, in mM: 154 NaCl, 5.6 KCl, 3.5 NaHCO_3_, 5.6 glucose, and 5 HEPES. Brain tissues were gently removed using forceps and scissors, and the cerebral cortex was carefully dissected and transferred to a Petri dish containing ice-cold Neurobasal medium supplemented with 5 mL L-glutamine, 50 IU/mL penicillin, 50 µg/mL streptomycin, 10 mg/mL gentamicin, and 10% fetal bovine serum (FBS). Cortical tissue fragments were dissociated in 2 mL Neurobasal medium containing 10% FBS by gentle trituration using a Pasteur pipette. Viable cells were counted using a Neubauer chamber under a Leica 6S E microscope (Wetzlar, Germany) after trypan blue staining. Cells were seeded at a density of 6 × 10^4^ cells per well in 96-well plates previously coated overnight with 5% poly-D-lysine. Cultures were maintained at 37 °C in a humidified atmosphere containing 5% CO_2_/95% air. After 1.5 h, the culture medium was replaced with serum-free Neurobasal medium supplemented with 2% B-27 supplement [39]. Every three days, 50% of the culture medium was replaced with fresh medium containing the same supplement until the day of the experiment. MTT experiments under toxic conditions were performed between days 6 and 8 in culture (DIV6–8) [40,41].

For each independent neuronal culture, cells were distributed among the different experimental conditions so that, whenever possible, untreated control, toxic-stimulus, and treatment groups were represented within the same neuronal preparation, thereby minimizing variability between independent cultures. No formal randomization or blinding procedure was applied during treatment administration or data acquisition. Nevertheless, all experimental groups were processed in parallel under identical culture and assay conditions and according to the same predefined experimental procedures.

Wells were excluded only in cases of evident technical failure, culture contamination, or abnormal culture conditions, and no experimental data were excluded on the basis of the magnitude or direction of the observed treatment effect. No formal a priori power calculation was performed. The number of experimental replicates was based on independent primary neuronal preparations and replicate wells, taking into account the biological variability inherent to primary neuronal cultures and previous experience with the same experimental model, while following the principles of reduction and refinement in animal experimentation. The number of replicate wells (n) and independent neuronal cultures (N) used in each experiment is indicated in the corresponding figure legends.

Animal-related experimental reporting was revised in accordance with the ARRIVE 2.0 recommendations, including reporting of experimental units, sample size, allocation to experimental groups, inclusion and exclusion criteria, and randomization and blinding procedures where applicable.

### 4.5. Measurements of Relative [Ca^2+^]_c_ with Fluo-4-AM

These experiments were performed using the fluorescent probe Fluo-4-AM (Thermo Fisher Scientific, Waltham, MA, USA) and a FLUOstar Optima microplate reader (BMG Labtech, Offenburg, Germany). After removal of the culture medium, cells were incubated for 45 min at 37 °C in the dark in a loading solution containing (in mM): 5.9 KCl, 144 NaCl, 1.2 MgCl_2_, 11 glucose, and 10 HEPES/NaOH (pH 7.4), supplemented with 10 µM Fluo-4-AM and 0.2% Pluronic acid. After loading, cells were washed twice in the dark with Krebs-HEPES buffer at room temperature. Fluorescence recordings were performed at room temperature in Krebs-HEPES buffer. The same extracellular solution was used during stimulation with caffeine (20 mM) and histamine (100 µM), which were added directly to the recording medium. For depolarization with high K^+^, extracellular KCl was increased to 35 mM; the 35 mM K^+^ solution was prepared by increasing KCl from 5.9 to 35 mM while reducing NaCl by an equimolar amount to maintain osmolarity. Cells were preincubated with the indicated concentrations of marjoram extract for 5 min before stimulation, and the extract remained present during recording. Fluorescence was excited at 488 nm and emission was recorded at 522 nm. At the end of each experiment, cells were incubated for 10 min with Triton X-100 (5%) to determine F_max_ and then with MnCl_2_ (2 mM, 10 min) to determine F_min_ [42]. A stable baseline was recorded for 30 s before stimulation, and F_basal_ was calculated as the mean fluorescence during this prestimulus period. Changes in the relative cytosolic Ca^2+^ signal were expressed as normalized Fluo-4 fluorescence according to F_x_ = (F_measured_ − F_basal_)/(F_max_ − F_min_) × 100. For quantitative analysis, the stimulus-evoked response was defined as the maximum fluorescence peak after stimulus application. All experiments were performed in triplicate at room temperature using cells 1–3 days after culture.

### 4.6. Electrophysiological Recording and Data Analysis

Voltage-clamp and current-clamp recordings were obtained using the whole-cell and perforated-patch configurations, respectively, of the patch-clamp technique. Recordings were made using patch pipettes of thin fire-polished borosilicate glass (Kimax 51, Witz Scientific, Heijningen, The Netherlands) to obtain a final series resistance of 5–7 MΩ when filled with the standard intracellular solutions and mounted on the headstage of an EPC-9 patch-clamp amplifier using the PatchMaster software (version 2.91) (HEKA Electronic, Lambrecht, Germany). To establish the perforated patch configuration, we used a pipette solution containing 50–100 ng/mL amphotericin B. Amphotericin B was dissolved in dimethyl sulfoxide and stored at −20 °C in stock aliquots of 50 µg/mL. Fresh pipette solution was prepared every 2 h. Recording started when the access resistance decreased below 20 MΩ. Series resistance was compensated by 80% and monitored throughout the experiment.

For the Ca^2+^ (*I*_Ca_), Na^+^ (*I_Na_*) and K^+^ (*I_K_*) currents and for the current-clamp recordings, data were acquired with a sample frequency ranging between 5 and 10 kHz and filtered at 1–2 kHz. Recording traces with leak currents at >100 pA (voltage-clamp) or >10 pA (current clamp) or a series resistance of >20 MΩ were discarded. During the seal formation with the patch pipette, the chamber was continuously perfused with a Tyrode solution containing (in mM): 137 NaCl, 5 KCl, 1 MgCl_2_, 2 CaCl_2_, 10 HEPES/NaOH (pH 7.4). Once the patch membrane was perforated and the whole-cell configuration of the patch-clamp technique was established, the cell was constantly superfused with the Tyrode solution, but containing nominally 0 mM Ca^2+^ (to measure *I_Na_*), 2.5 mM Ca^2+^ (to measure *I_K_*_Ca_), 2.5 mM Ca^2+^ (to measure *I_K_*_v_) or 10 mM Ca^2+^ + 1 µM tetrodotoxin (TTX) to prevent the activation of the Na^+^ current (to measure *I*_Ca_) (see Section 2.2 to 2.5 for specific experimental protocols). For *I_Na_* and *I*_Ca_ recordings, cells were dialyzed with an intracellular solution containing (in mM): 10 NaCl, 100 CsCl, 14 EGTA, 20 TEA-Cl, 5 Mg-ATP, 0.3 Na-GTP and 20 HEPES/CsOH (pH 7.4 with CsOH). To record *I_K_* and current clamp, CsCl and TEA-Cl were replaced by KCl (pH 7.4 with KOH). External solutions were exchanged using a pump perfusion system (2 mL/min), allowing the perfusion solution to be changed within 20 s.

### 4.7. Measurement of MTT-Reducing Activity in Bovine Chromaffin Cells and Cortical Neurons

MTT-reducing activity was evaluated using the MTT colorimetric assay [43] in bovine chromaffin cells and cortical neurons under two toxic paradigms: veratridine-induced Ca^2+^ overload and oligomycin/rotenone-induced mitochondrial stress. Bovine chromaffin cells were used 48 h after plating, whereas primary cortical neurons were studied between DIV6 and DIV8. Cells were seeded on 96-well plates. Bovine chromaffin cells were seeded at 2 × 10^5^ cells per well whereas cortical neurons were seeded at 6 × 10^4^ cells per well. Cells were incubated for 24 h with the indicated marjoram extract concentrations, after which veratridine (30 µM) or oligomycin/rotenone (10 and 30 µM, respectively) was added for a further 24 h. The extract remained present during toxin exposure. The endpoint was defined as a priori as MTT-reducing activity and not as a direct measure of cell number, membrane integrity, cytoprotection, or neuroprotection.

MTT (0.5 mg/mL) was added to the wells and incubated for 15 min at 37 °C. Formazan was solubilized with DMSO and absorbance was measured at 540 nm using a FLUOstar Optima reader (BMG Labtech, Ortenberg, Germany). Results were expressed as percentage of MTT-reducing activity relative to the corresponding untreated control, set to 100%. Replicate wells were performed within each independent culture. For inferential statistics, replicate wells from the same culture and experimental condition were averaged to obtain one value per culture, and the independent culture was used as the biological unit. The original experimental series did not include extract-only groups, concentration-matched vehicle-only controls for every extract condition, cell-free wells containing extract plus MTT reagents, a separate test of absorbance interference at 540 nm, or an independent orthogonal assay of cell number, membrane integrity, apoptosis, or necrosis. These limitations are therefore taken into account in the interpretation of the MTT data throughout the manuscript.

### 4.8. Chemicals

Salts to make the saline solutions were obtained from Merck (Madrid, Spain). Collagenase type I was from Roche (Madrid, Spain), while DMEM, fraction V fetal bovine albumin, penicillin-streptomycin were from Gibco (Madrid, Spain). Fluo-4-AM was obtained from Molecular Probes (Life Technologies, cat. no. F14217, Carlsbad, CA, USA); veratridine, oligomycin and rotenone were obtained from Sigma-Aldrich (Madrid, Spain); and the rest of the chemical reagents and solutions were from Merck and Panreac Chemical (Barcelona, Spain). Phenolic compounds were purchased from different suppliers (Sigma-Aldrich, Madrid, Spain; Phytolab, Vestenbergsgreuth, Germany; and Extrasynthese, Genay, France).

### 4.9. Statistical Analysis

Data are presented as representative original recordings or as pooled quantitative data. For transparency, the total number of individual experimental determinations (cells or wells) is reported as n, whereas the number of independent primary cultures is reported as N. The independent primary culture was considered the biological experimental unit for all inferential statistical analyses. When several cells or wells were obtained from the same culture under the same experimental condition, these within-culture determinations were averaged to obtain a single culture-level value for that condition. The experimental designs were classified before analysis as paired two-condition designs or within-culture repeated-measures designs; no independent-samples Mann–Whitney U tests or ordinary one-way ANOVA were used in the final culture-level analysis. Student’s paired *t*-test was used only for a single prespecified two-condition within-culture comparison (e.g., control vs. marjoram for resting membrane potential). When three or more concentrations or sequential treatment conditions were evaluated within the same cultures, one-way repeated-measures ANOVA was used, followed, when planned contrasts were required, by Holm correction for multiple comparisons. Pharmacological-control contrasts that were specified separately from the concentration series were also Holm-corrected within the corresponding family of comparisons. The use of parametric tests was based on the continuous nature of the measurements and on inspection of the culture-level values and paired differences for marked asymmetry or influential outliers; given the small number of independent cultures in several experiments, formal normality tests were not used as the sole criterion because of their limited power. If a dataset showed a clear departure from these assumptions, the prespecified alternatives were the Wilcoxon matched-pairs test for two-condition paired designs and the Friedman test for repeated-measures designs. No such departure requiring replacement of the reported parametric analysis was identified in the datasets analyzed here. Effect sizes are reported as Cohen’s dz for paired comparisons and partial η^2^ for repeated-measures ANOVA, with 95% confidence intervals for paired mean differences whenever estimable. Exact *p* values are reported where possible; all *p* values associated with multiple planned comparisons are multiplicity-adjusted, and *p* < 0.05 was considered statistically significant. Analyses were performed using GraphPad Prism version 8.01 (GraphPad Software, La Jolla, CA, USA).

## 5. Conclusions

In conclusion, the culture-level analysis supports a complex modulatory effect of *Origanum majorana* extract on the electrical behavior of bovine chromaffin cells. Significant effects were retained for voltage-dependent Ca^2+^ and Na^+^ currents at the highest concentration tested, for the overall modulation of both putative Ca^2+^-dependent and voltage-dependent K^+^ current components, and for membrane depolarization. These findings indicate a substantial effect of marjoram extract on membrane excitability, while the relatively small number of independent cultures should be considered when interpreting the precision of the estimates.

The Ca^2+^-imaging experiments showed lower mean K^+^-evoked responses and higher mean caffeine-evoked responses in the presence of marjoram extract, whereas histamine-evoked responses were unchanged. However, the K^+^ and caffeine concentration effects did not remain statistically significant after culture-level analysis and multiplicity correction. The Ca^2+^-imaging findings are best regarded as directional associations compatible with differential effects on plasma-membrane Ca^2+^ entry and ryanodine-sensitive intracellular stores, rather than as evidence that marjoram directly activates or sensitizes RyR receptors.

The MTT experiments showed statistically significant preservation of MTT-reducing activity only in selected conditions, with the most consistent effects observed in bovine chromaffin cells at the lower marjoram concentrations. In primary cortical neurons, the marjoram comparisons did not remain significant after multiplicity correction. Because extract-only, matched vehicle, cell-free interference, and orthogonal assays of cell number or membrane integrity were not included, these findings are restricted to the MTT endpoint and do not establish cytoprotection, neuroprotection, or antioxidant activity.

Overall, the present findings are correlative. Although *O. majorana* extract modulates membrane excitability and is associated with changes in intracellular Ca^2+^ handling and MTT-reducing activity, a causal contribution of ryanodine-sensitive intracellular stores to the preservation of cellular metabolic activity remains to be established. Further studies using assay-interference controls, complementary assays of cell number, membrane integrity, apoptosis, or necrosis, experiments combining marjoram with an RyR inhibitor under matched vehicle conditions to assess the contribution of ryanodine-sensitive intracellular stores, and fractionated or isolated extract constituents will be required to establish the biological meaning and mechanisms underlying these observations.

## Figures and Tables

**Table 1:** Phenolic compound composition (mg/g dry extract) of the marjoram extract obtained by pressurized liquid extraction (PLE).

Compound	mg/g Extract
3,4-dihydroxyphenyllactic acid ^a^	0.11 ± 0.01
Neochlorogenic acid *	0.21 ± 0.03
Protocatechuic acid *	0.24 ± 0.02
Cryptochlorogenic acid *	0.67 ± 0.03
Vicenin 2 *	2.22 ± 0.06
Caffeic acid hexoside ^a^	0.13 ± 0.01
Caffeic acid *	0.84 ± 0.02
Luteolin-*C*-hexoside ^a^	1.25 ± 0.12
6-Hydroxyluteolin-7-*O*-glucoside ^a^	40.34 ± 3.07
Luteolin rutinoside ^a^	1.55 ± 0.08
Luteolin-*O*-glucoside ^a^	21.97 ± 1.55
Luteolin-7-*O*-glucoside *	16.78 ± 0.94
Luteolin-7-*O*-glucuronide *	3.52 ± 0.28
Diosmin *	5.91 ± 0.35
Apigenin-7-*O*-glucoside *	2.71 ± 0.13
Apigenin-7-*O*-glucuronide *	2.56 ± 0.17
Rosmarinic acid *	36.17 ± 1.40
Lithospermic acid *	9.02 ± 0.37
Lithospermic acid isomer I ^a^	22.14 ± 0.71
Salvianolic acid B *	2.05 ± 0.06
Lithospermic acid isomer II ^a^	2.10 ± 0.08
Salvianolic acid isomer I ^a^	3.79 ± 0.12
Sagecoumarin isomer ^a^	0.74 ± 0.04
Eriodictyol *	0.59 ± 0.02
Luteolin *	1.24 ± 0.06
Quercetin *	0.38 ± 0.02
Salvianolic acid isomer II ^a^	1.70 ± 0.11
Hydroxydimethoxy flavone ^a^	1.02 ± 0.07
Rosmarinic acid derivative I ^a^	0.23 ± 0.01
Rosmarinic acid derivative II ^a^	0.33 ± 0.06
Trihydroxytrimethoxy flavone ^a^	0.90 ± 0.05
Naringenin *	1.20 ± 0.05
Apigenin *	0.31 ± 0.02
Trihydroxydimethoxy flavone I ^a^	0.89 ± 0.05
Trihydroxydimethoxy flavone II ^a^	0.74 ± 0.03
Hesperetin *	0.20 ± 0.01
Dihydroxydimethoxy flavone ^a^	0.07 ± 0.01

* Identification confirmed by comparison with an authentic standard. ^a^ Tentatively identified according to UV–Vis spectra and MS/MS fragmentation profiles, as previously described [17,18]. Values are expressed as mean ± S.D. of three replicates.

## Data Availability

The original contributions presented in this study are included in the article and Supplementary Material. Further inquiries can be directed to the corresponding author.

## Supplementary Materials

The following supporting information can be downloaded at https://www.mdpi.com/article/10.3390/ph19091493/s1, S1: Technical specification sheet for imported marjoram leaves (*Origanum majorana*).
