# Supplementary material for: Marjoram Extract Modulates Ion Currents, Intracellular Calcium Signaling, and MTT-Reducing Activity in Bovine Chromaffin Cells and Rat Cortical Neurons
Source: Pharmaceuticals (Basel). 2026 Sep 19;19(9):1493. doi: 10.3390/ph19091493 (PMC13610231; doi:10.3390/ph19091493)
Supplement: Supplementary file 1 [file pharmaceuticals-19-01493-s001.zip › pharmaceuticals-4475508-supplementary S1. Technical specification sheet for imported marjoram leaves (Origanum majorana).pdf]

MURCIANA DE HERBORISTERIA S.A.
R.S. 2400651 - MU
R.S. 2501235 - MU
C/. Mar Mediterráneo, 6 • Tel. 968 86 18 72 • Fax 968 86 11 02
www.muherb.com • e-mail: info@muherb.com
30163 COBATILLAS - Murcia (España)

TECHNICAL SPECIFICATION SHEET

MARJORAM LEAVES IMPORT

Revision: 9

Date: 27/06/2025

**Product**	Imported marjoram leaves
**Description**	Healthy, clean and dried leaves and flowering tops of *Origanum majorana*
**Organoleptic characteristics**	
▪ Appearance	Compliant
▪ Odour	Characteristic, free from foreign odours
▪ Colour	Green
**Physicochemical characteristics**	
Moisture (%)	15.00 max.
Total ash (%)	16.00 max.
Insoluble ash (%)	4.00 max.
Essential oil (%)	0.50 min.
**Microbiological characteristics**	
Escherichia coli (CFU/g)	≤ 100
Salmonella spp. (absence or presence)	Absent / 25 g
Listeria monocytogenes (CFU/g)	≤ 100
**Foreign matter**	
≤ 5% stems and floral remains and ≤ 2% other foreign matter	
**Pesticide residues and contaminants**	
Murciana de Herboristería, S.A., as established in its Quality System, monitors the maximum permitted levels of pesticides and other contaminants in its products in compliance with European Regulations.	
**Nutritional information (per 100 g)**	
Energy value (kJ/kcal)	1,140 / 271
Fat (g)	7.0
Saturated fatty acids (g)	0.5
Protein (g)	12.7
Carbohydrates (g)	60.6
Sugars (g)	4.1
Salt* (mg)	192.5
*The salt content corresponds exclusively to the sodium naturally present in the food.<br>Nutritional values have been obtained from publicly accessible databases of various official bodies and should be considered indicative. Actual values may vary depending on the different varieties, origins, cultivation and harvesting conditions, climates, etc.	
**Please note**	
The information stated in this technical specification sheet is general for the product. Always verify the analytical data of the supplied batch, such as microbiological parameters, pesticides, contaminants and allergens, in the batch certificate of analysis.	

ALLERGEN DECLARATION

Information provided by our suppliers may be subject to change and should therefore always be checked against the analysis or other supporting documentation provided by the supplier.

ALLERGENIC PRODUCT: the allergen forms part of the product composition.
CROSS-CONTAMINATION: allergens may be present due to possible cross-contamination during cultivation, at the original supplier's facilities or during transport.
ABSENCE: when the product is not allergenic in itself and there is no possibility of cross-contamination.

ALLERGENS	ALLERGENIC PRODUCT	CROSS-CONTAMINATION	ABSENCE
Cereals containing **gluten**: wheat, rye, barley, oats or their hybridised strains and products thereof			X
**Crustaceans** and products thereof			X
**Eggs** and products thereof			X
**Fish** and products thereof			X
**Peanuts** and products thereof			X
**Soybeans** and products thereof			X
**Milk** and products thereof (including lactose)			X
**Nuts**: almonds, hazelnuts, walnuts, cashews, pecans, Brazil nuts, pistachios, macadamia nuts or Queensland nuts and products thereof			X
**Celery** and products thereof		X	
**Mustard** and products thereof			X
**Sesame seeds** and products thereof			X
**Sulphur dioxide and sulphites** at concentrations > 10 mg/kg in terms of total SO_2			X
**Lupin** and products thereof			X
**Molluscs** and products thereof			X

**Packaging and auxiliary material**	The packaging and auxiliary materials used are suitable for food use and comply with European Regulations.
**Product presentation**	Whole, cut or ground.
**Best before**	Maximum 4 years from the date of manufacture.
**Storage conditions**	Store in a cool, dry place. Do not expose directly to sunlight. Keep away from products that give off strong odours.
**Directions for use**	Before consumption, heat-treat for at least 15 seconds at 75 °C.

Additional information

NON-GMO: products supplied by Murciana de Herboristería, S.A. are not derived from GMO raw materials.

NON-IRRADIATED: products supplied by Murciana de Herboristería, S.A. have not been subjected to ionising radiation.

Infestation:

1. With respect to coriander seeds, fennel seeds, roots, chamomile flowers and flowers in general, we cannot guarantee that the product will remain in perfect condition throughout its shelf life, as it will be exposed to infestation unless specifically vacuum-treated or heat-treated. Even after treatment, treated products may become reinfested if stored incorrectly. Seeds, herbs and spices treated in any form with authorised chemicals are still susceptible to possible infestation, as insect eggs are not destroyed by current chemical processes.

2. We guarantee that all products are shipped treated/disinfested from our facilities.

Use in production: if the items or any part thereof supplied under the contract and accompanying documentation are processed, altered or handled in any way by the buyer / recipient of the goods or any other person, the quality of the goods shall be deemed acceptable to the buyer. All customer quality checks must be completed on all items prior to production and use.
